# New Avenues for the Treatment of Huntington’s Disease

**DOI:** 10.3390/ijms22168363

**Published:** 2021-08-04

**Authors:** Amy Kim, Kathryn Lalonde, Aaron Truesdell, Priscilla Gomes Welter, Patricia S. Brocardo, Tatiana R. Rosenstock, Joana Gil-Mohapel

**Affiliations:** 1 Island Medical Program and Faculty of Medicine, University of British Columbia, Victoria, BC V8P 5C2, Canada; amyjukim@gmail.com (A.K.); klalonde@alumni.ubc.ca (K.L.); 2 Division of Medical Sciences, University of Victoria, Victoria, BC V8P 5C2, Canada; atruesdell2020@meds.uwo.ca; 3 Schulich School of Medicine and Dentistry, Western University, London, ON N6A 5C1, Canada; 4 Neuroscience Graduate Program, Federal University of Santa Catarina, Florianópolis 88040-900, Brazil; prii.gomesw@hotmail.com (P.G.W.); patricia.brocardo@ufsc.br (P.S.B.); 5 Institute of Cancer and Genomic Science, College of Medical and Dental Sciences, University of Birmingham, Edgbaston, Birmingham B15 2TT, UK; tatirosenstock@gmail.com; 6 Department of Pharmacology, University of São Paulo, São Paulo 05508-000, Brazil

**Keywords:** autophagy, clinical trial, disease-modifying treatment, epigenetics, genetics, Huntington’s disease, pre-clinical study, transgenic mouse model, trophic support

## Abstract

Huntington’s disease (HD) is a neurodegenerative disorder caused by a CAG expansion in the HD gene. The disease is characterized by neurodegeneration, particularly in the striatum and cortex. The first symptoms usually appear in mid-life and include cognitive deficits and motor disturbances that progress over time. Despite being a genetic disorder with a known cause, several mechanisms are thought to contribute to neurodegeneration in HD, and numerous pre-clinical and clinical studies have been conducted and are currently underway to test the efficacy of therapeutic approaches targeting some of these mechanisms with varying degrees of success. Although current clinical trials may lead to the identification or refinement of treatments that are likely to improve the quality of life of those living with HD, major efforts continue to be invested at the pre-clinical level, with numerous studies testing novel approaches that show promise as disease-modifying strategies. This review offers a detailed overview of the currently approved treatment options for HD and the clinical trials for this neurodegenerative disorder that are underway and concludes by discussing potential disease-modifying treatments that have shown promise in pre-clinical studies, including increasing neurotropic support, modulating autophagy, epigenetic and genetic manipulations, and the use of nanocarriers and stem cells.

## 1. Introduction

Huntington’s disease (HD) is a rare, inherited, neurodegenerative condition that causes progressive motor deficits, psychiatric symptoms, and cognitive impairment. The diagnosis of HD can be made anytime during an individual’s life, but in most cases, this occurs in middle adulthood [1]. HD is a progressive disease, and patients in the early stages may experience subtle involuntary movements, slight difficulty with executive functions, and depressed mood [2,3]. Despite this, these individuals generally remain independent. As the disease progresses, patients begin to require assistance. They may no longer be able to drive or remain employed. Problem solving and coordination become increasingly difficult, and falls start to become more frequent. During the late stages, HD patients may become bedridden, require feeding tubes, and be unable to speak due to loss of voluntary motor control and increased involuntary movements [2]. In addition, at this stage in disease progression, dementia is often severe and can affect all aspects of cognition [4]. Death occurs at a median of 18 years after symptom onset, with infections, specifically aspiration pneumonia, being the most common cause of death [5,6].

Worldwide, the prevalence of HD is estimated to be 2.7 per 100,000 individuals [7]. However, it is known that this varies regionally. Western populations such as Canada, the United States, the United Kingdom, and Australia tend to have the highest prevalence, while Asian countries such as Japan, Korea, Taiwan, and Hong Kong tend to have a lower prevalence of the disease [8,9]. The most recent studies have estimated the prevalence of HD among the general population in British Columbia, Canada, to be 13.7 per 100,000 individuals, which is slightly lower than the general Caucasian population (estimated as 17.2 per 100,000 individuals) [10].

HD is inherited in an autosomal dominant manner and is caused by an expansion of a cytosine-adenine-guanine (CAG) trinucleotide repeat in the coding region of the HD gene, located in the short arm of chromosome 4 (4p16.3) [11]. The HD gene encodes the protein huntingtin (HTT), which is found in many tissues throughout the body, including throughout the central nervous system (CNS). While its function has not been entirely elucidated, it has been proposed to play critical roles in several cellular events such as protein trafficking, transport of vesicles, and selective autophagy [12,13]. When the CAG sequence expands beyond the normal range of 6–26 repeats, it becomes unstable and may expand further in subsequent generations, especially with paternal transmission. The intermediate range is between 27 and 35 repeats, and individuals rarely exhibit the clinical phenotype. The threshold for developing HD is commonly considered 36 repeats and above, although full penetrance is not seen until 40 repeats are present [14,15]. Higher numbers of CAG repeats are also thought to be associated with an earlier onset of disease, a faster rate of clinical progression, and increasing disease severity [16]. In addition, other factors, namely, additional genetic predispositions and/or environmentally induced modifications, may play a role in HD etiology and progression [17,18].

With this increase in the number of CAG repeats (which results in an extended polyglutamine expansion in the N-terminal region of the protein), HTT becomes prone to misfolding and subsequent formation of insoluble aggregates that are often found post-mortem both in the nucleus (forming neuronal intranuclear inclusions, NIIs) and cytoplasm of neurons [19,20]. These aggregates accumulate and lead to cell dysfunction and apoptosis, eventually causing severe atrophy of the affected brain areas [21]. Initially, these deleterious aggregates seem to primarily affect the caudate nucleus, putamen, nucleus accumbens, and olfactory tubercule, which together comprise the striatum. This atrophy of the striatum constitutes the neuropathological hallmark of HD [22]. Aside from the striatum, atrophy is also observed in the cerebral cortex, cerebellum, hypothalamus, hippocampus, and select nuclei within the thalamus and brainstem [22,23,24,25,26,27,28].

The symptoms of HD, which usually arise between 30 and 50 years of age, fall under three main domains: motor, cognitive, and psychiatric [29,30]. As mentioned above, these symptoms often begin insidiously and are progressive. Initially, they may even go unrecognized by the patient for several years prior to diagnosis [31].

One of the key motor symptoms of HD is chorea, which is defined as brief, involuntary movements that generally affect the trunk, face, and arms. Chorea is progressive and results in difficulty with voluntary movements, eventually affecting walking, speaking, and swallowing. Other motor symptoms include bradykinesia, dystonia, hyperreflexia, and slowing of eye saccades [32]. These motor symptoms are closely related to impairment in the modulation of the motor cortex by the direct and indirect pathways of the basal ganglia. The gamma-aminobutyric acid (GABA) ergic neurons of the direct pathway preferentially use substance P as their co-neurotransmitter, whereas the neurons of the indirect pathway use enkephalin [33]. Early in HD, the neurons from the indirect pathway are disproportionately more affected than those from the direct pathway. Since the indirect pathway normally suppresses involuntary movements, it is believed that this imbalance leads to the typical chorea observed in HD patients. However, as the disease progresses, neurons in the direct pathway are also affected, which is thought to explain the increasing bradykinesia and rigidity observed in advanced-stage HD patients [30,34,35,36].

Cognitive deficits in HD generally manifest as a decline in executive function, with patients frequently having increasing difficulty with attention, concentration, multi-tasking, and decision making. Depression and loss of memory and insight are also commonly observed [3,37,38]. The psychiatric symptoms of HD are broad, and prior to the onset of motor symptoms, patients may be mistakenly diagnosed with a primary psychiatric disorder. Anxiety, obsessive-compulsive disorder, mania, and psychotic symptoms, including hallucinations and delusions, may also be observed, but depression, irritability, and increased impulsivity are most commonly reported [39]. Suicide rates in HD are also significantly higher than those of the general population at 7% [40]. The basis of the cognitive and psychiatric symptoms has yet to be fully elucidated, but striatal dopaminergic dysregulation is thought to play a role. Recent research has also shown that atrophy and impairments in synaptic plasticity within the fronto-striato-limbic loop are potentially implicated in both the cognitive and psychiatric symptoms seen in HD [41,42].

While unrelated to the three domains mentioned above, weight loss and cachexia are also frequently observed [31]. Human HD patients and HD mouse models have both been demonstrated to exhibit hypermetabolic states and negative energy balances. This is thought to result from early hypothalamic atrophy, leading to a dysregulation of hormones including orexin, somatostatin, and ghrelin [26,43,44].

### 1.1. Mechanisms of Neurodegeneration in HD

There are several mechanisms by which mutant huntingtin (mHTT) is thought to exert its deleterious effects in HD patients. These have been elucidated through numerous in vitro, in vivo, and post-mortem studies conducted over the last two decades. These pathological processes include, but are not restricted to, N-Methyl-D-Aspartate (NMDA) receptor-mediated excitotoxicity, dopaminergic dysfunction, mitochondrial dysfunction and oxidative stress, dysregulated autophagy, abnormal protein aggregation, and disrupted gene transcription, as well as loss of trophic support. In the HD brain, these abnormalities are thought to be somewhat linked to both a toxic gain of function of mHTT and a loss of function of the normal HTT [30,45,46,47,48]. Although a detailed review of these multiple mechanisms is outside of the scope of the present review, the contributions of excitotoxicity (i.e., dysfunction of the cortico-striatal pathway), dopaminergic toxicity (i.e., dysfunction of the nigrostriatal pathway), and dysfunction of mitochondrial and autophagic pathways, as well as reduced trophic support, are briefly summarized in the sub-sections below (for a more in-depth review, please see [30]).

#### 1.1.1. Excitotoxicity

Striatal neurodegeneration in HD primarily affects the GABAergic medium spiny neuron (MSN) population, which corresponds to roughly 85% of the striatum. The remaining striatal neurons include interneurons, which are relatively spared from neurodegeneration in HD [49]. Neurotransmitter expression among the striatal interneuron population is heterogeneous and can include somatostatin, neuropeptide Y, nicotinamide adenine dinucleotide phosphate (NADPH) diaphorase, calretinin, and acetylcholine [50]. It is hypothesized that the selective degeneration of the GABAergic MSN population may be due to NMDA receptor-mediated excitotoxicity. Corroborating this theory, it is known that MSNs receive substantial glutamatergic excitatory input from the cortex (through the cortico-striatal pathway). In addition, MSNs expressed higher levels of NMDA receptors (particularly the subunit NR2B) when compared to striatal interneurons [51], which makes them more susceptible to excitotoxicity. Of note, the NR2B subunit confers greater permeability to the NMDA receptor and lowers its deactivation time [52], which, in turn, can render MSNs more susceptible to glutamate-mediated excitotoxicity when compared with striatal interneurons. Supporting such a hypothesis, in vitro studies have shown increased excitotoxic cell death when mHTT was co-expressed with NR1A/NR2B-type NMDA receptors [53]. Furthermore, in vivo studies have shown increased selective degeneration of MSNs in a murine model of HD (Hdh(CAG)150) crossed with a transgenic model that overexpresses the NR2B subunit [54].

Further supporting the role of glutamate-mediated excitotoxicity in HD, elevated levels of glutamate have been found in the cortex of post-mortem human HD brains [55]. This may be a result of impaired astrocyte function, as decreased levels of astrocytic glutamate transporter (EAAT1) and glutamate transporter 1 (GLT1) mRNA have been observed in the neostriatum of post-mortem human HD brains [56]. In agreement, decreased GLT1 mRNA levels and reduced glutamate uptake have also been seen in transgenic HD mouse models (R6/1 and R6/2 mice) as well as cultured astrocytes expressing an N-terminal fragment of mHTT [57,58]. In addition, an increased release of astrocytic glutamate into the extracellular space has also been demonstrated in bacterial artificial chromosome (BAC) HD mice [59].

Microglia may also contribute to the excitotoxicity observed in HD through increased activity of the kynurenine pathway. This pathway produces excessive amounts of quinolinic acid (QA) and 3-hydroxykynurenine (3HK) in HD. QA is a potent, selective NMDA receptor agonist, while 3HK is thought to be a potentiator of QA-induced damage [60,61].

Together, excessive activation of NMDA receptors (as a result of augmentation in the release of glutamate from cortical afferents, diminishment in glutamate uptake by glial cells, an increase in the sensitivity of these receptors on post-synaptic striatal projections, and enhanced production of QA) is thought to result in changes in intracellular calcium homeostasis and mitochondrial function, which will ultimately lead to neuronal degeneration and cellular death [30,62].

#### 1.1.2. Dopaminergic Dysfunction

Dopaminergic projections from the substantia nigra pars compacta (SNc) to the striatum are also dysfunctional in HD. Studies in human HD brains have shown increased dopamine (DA) levels during the early stage of disease progression and depleted levels during the late stage [63,64]. These DAergic alterations that affect the nigrostriatal pathway are thought to contribute to the early (hyperkinesia) and late (akinesia) phenotypes seen in HD patients and have led to the hypothesis that DA levels are biphasic in HD and any treatments targeting these may have benefits in HD patients [65,66].

DA also contributes to the formation of reactive oxygen species (ROS), which, in turn, may lead to the activation of the pro-apoptotic c-Jun N-terminal kinase (JNK)/c-Jun pathway (through synergistic interaction with mHTT) [67] and inhibition of the autophagocytic clearance of mHTT [68]. In addition, DA itself can be a source of ROS, as this neurotransmitter can undergo both enzymatic and non-enzymatic degradation, with the former producing 3,4-dihydrophenylacetic acid (DOPEC) and hydrogen peroxide (H_2_O_2_) and the latter producing DA quinones and H_2_O_2_ along with DA semiquinones and superoxide (O_2_^−^). H_2_O_2_ and O_2_^−^ can become further oxidized, resulting in the production of a highly reactive hydroxyl radical (HO·) [69]. This DA-induced increase in ROS may contribute to cell death via misfolding and aggregation of proteins, lipoperoxidation of membranes, and organelle leakage [70].

#### 1.1.3. Mitochondrial Dysfunction and Oxidative Stress

Mitochondrial dysfunction in HD goes far beyond changes in glycolysis and oxidative stress; it also includes modifications in the oxygen consumption rate, alterations in the various enzymatic complexes of the electron transport chain, and changes in mitochondrial transcription regulation as well as in mitochondrial biogenesis, turnover, and degradation (through a process called mitophagy). Notably, all these pathways can be regulated directly and indirectly by mHTT [71]. In support, an increase in mitochondrial fragmentation has been observed both in the brain and lymphoblasts of HD patients [72,73]. This was shown to be a consequence of mHTT binding dynamin-related protein 1 (DRP1) tighter than its wild-type counterpart and resulting in a higher ratio of mitochondrial fission to fusion [74].

This imbalance between fission and fusion can limit osmotic homeostasis and mitochondrial motility, decrease energy production, increase oxidative stress, and impair Ca_2_^+^ buffering, resulting in a net effect of increased neuronal death [75,76]. mHTT also interacts with peroxisome proliferator-activated receptor-g coactivator-1 alpha (PGC-1α), a nuclear transcriptional co-regulator known as the master controller of mitochondrial biogenesis. mHTT has been shown to bind the PGC-1α promoter and interfere with cAMP response element-binding protein (CREB)/TATA-box binding protein associated factor 4 (TAF4), leading to decreased expression of this transcriptional regulator [77]. Of note, extra-synaptic NMDA receptor stimulation of mHTT-expressing neurons has also been shown to increase neuronal death by impairing this CREB-PGC-1α cascade and increasing Rhes (Ras homolog enriched in the striatum), a protein known to interfere with mHTT aggregation [78] and activate autophagy [79]. The downstream roles of PGC-1α are numerous and include mitochondrial biogenesis, adaptive thermogenesis, antioxidant defense, metabolism of glucose/fatty acids, and oxidative phosphorylation, among others [80]. In fact, it has been demonstrated that not only does PGC-1α effectively suppress the production of free radicals, but it is also required for adaptive thermogenesis as a co-activator of uncoupling protein 1 (UCP1) [81]. Unsurprisingly, increased ROS production and impaired adaptive thermogenesis have been well documented in murine models of HD [82,83,84]. Together, the consequences of mitochondrial dysfunction include impaired ATP production and oxidative damage to DNA, proteins, and lipids, as well as the activation of pro-apoptotic pathways [67,85,86]. Together, these effects culminate in increased neuronal death.

Interestingly, N171-82Q HD mice, which are known to display reduced body temperatures starting at 120 days of age, have been shown to live longer when housed at a higher ambient temperature (30 °C instead of 20 °C) [83]. Of note, although hypothermia had only been documented in some HD animal models [83,84], a recent study has now reported a case of hypothermia in a 29-year-old HD patient, who presented to the emergency department with a body temperature of 34 °C (axillary) [87]. These findings emphasize the fact that regular pathways of thermoregulation (such as those involving PGC-1α) may also be disrupted in HD and deserve further investigation.

#### 1.1.4. Autophagy Dysregulation

In addition to the processes described above, autophagy dysfunction has also been implicated in HD. While autophagic function typically declines with age [88], an mHTT-related decline in autophagy has also been shown to occur. Indeed, although HTT can be degraded by both the ubiquitin–proteasome system and autophagy when the protein is mutated, it undergoes post-transcriptional modifications that make it a more suitable target for the autophagic pathway [89]. However, as the disease progresses and mHTT accumulates into aggregates, the normal clearance pathways are unable to clear and degrade these aggregates [90,91]. Furthermore, the negative regulator of macroautophagy, mammalian target of rapamycin (mTOR), has been shown to be sequestered (and subsequently inhibited) within mHTT aggregates, leading to increased levels of macroautophagy [92]. However, the resulting autophagosomes have an impaired ability to recognize their cytosolic target cargo and remain relatively empty, leading to a net imbalance of energy expenditure and protein degradation [93]. These impairments in macroautophagy also lead to a build-up of intralysosomal lipofuscin, which interferes with the ability of the lysosomes to clear autophagic cargo [88].

HTT has also been shown to be a necessary scaffold protein for selective autophagy by regulating cargo recognition and autophagosome initiation. It is hypothesized that the expanded polyglutamine tract on mHTT may be responsible for the dysregulation of these processes [94]. Interestingly, significant alterations to chaperone-mediated autophagy (CMA) have also been reported in HD. One study using 111QHtt knock-in mice revealed that CMA is up-regulated in younger animals but subsequently drops at an accelerated rate in HD mice compared to age-matched controls [95]. This has led to the hypothesis that CMA is first up regulated to balance the macroautophagic impairment secondary to mTOR sequestration in mHTT aggregates as well as the putative dysregulation of the cargo recognition and autophagosome initiation processes within the selective autophagy system. This up-regulation eventually fails due to a combination of factors, including normal age-related decline and impaired lysosome function due to accumulation of lipofuscin. This failure of the autophagic system is thought to contribute to neuronal death in the HD brain [95,96]. In support, accumulation of morphologically defective mitochondria (which, in normal conditions, are cleared by autophagic mechanisms) has been observed in both patients and HD mouse models [97].

#### 1.1.5. Decreased Trophic Support

Brain-derived neurotrophic factor (BDNF) is a member of the neurotrophin family and a major regulator of synaptic plasticity, neuronal survival, and differentiation. Although widely expressed in the adult mammalian brain, BDNF is particularly abundant in the hippocampus and cerebral cortex [98,99,100]. BDNF is synthesized as a precursor protein, proBDNF, which is intra- and extracellularly cleaved to produce the mature active protein [101]. BDNF exerts its cellular effects through the actions of two receptors: tropomyosin receptor kinase B (TrkB) and p75 neurotrophin receptor (p75NTR) [102]. Most of its neuronal effects are mediated through its high affinity to TrkB [103,104]. However, BDNF can also interact with the low affinity p75NTR, which activates a different set of transduction cascades implicated in both pro- and anti-trophic processes such as neurite growth and apoptosis [105].

HTT is known to be a crucial regulator of BDNF transcription [106], and pathologic CAG expansions in the HD gene result in a reduction in BDNF function in HD, possibly because mHTT impairs both the transcription and the cortico-striatal transport of this neurotrophin, thus contributing to striatal neurodegeneration [29]. Indeed, HTT is part of the motor complex that drives anterograde and retrograde transport of BDNF-containing vesicles along microtubules. The binding of wild-type HTT to huntingtin-associated protein 1 (HAP1) indirectly regulates the assembly of dynactin, dynein, and kinesin 1 into the motor complex that controls vesicle transport. Conversely, mutant HTT inhibits the interaction between the motor complex proteins with microtubules, thus affecting BDNF vesicle transport along axonal microtubules [107]. In agreement, a recent report by Yu et al. (2018) [108] showed a slower travel time and decreased travel distance of BDNF-containing vesicles in zQ175 knock-in HD mice [108]. Furthermore, a significant reduction in BDNF gene transcription and protein levels has been found in multiple HD transgenic and knock-in mouse models [106,109,110,111,112,113,114,115,116]. More recently, a decrease in cortical BDNF release (and, consequently, in the availability of this neurotrophin in the striatum) has also been noted in zQ175 knock-in HD mice [108].

Moreover, a significant reduction in BDNF gene transcription and protein levels has also been found in HD-afflicted individuals [117,118]. Several studies have reported reduced BDNF protein levels in the striatum [107,119,120], cerebral cortex [106,120,121], cerebellum, and substantia nigra [120] of HD patients. Interestingly, these alterations were noted early in the disease progression, suggesting an early failure of trophic regulation in the neurodegenerative process [120]. Notably, reduced serum BDNF levels have also been reported in HD patients, and BDNF levels appeared to be inversely correlated with the number of CAG repeats and severity of illness [117,118,122]. Since BDNF crosses the blood–brain barrier [123,124], serum levels of this neurotrophin are thought to reflect its concentration within the brain [125] and measuring serum BDNF may provide a biomarker for the degenerative process occurring within the brain [126].

### 1.2. Animal Models of HD

To date, numerous animal models have been developed to study HD. Most of them have used rodents, particularly mice. Although both toxin-induced lesion models and genetic (transgenic and knock-in) models have been generated, given the genetic cause of HD, the latter are usually preferred, particularly when studying the effects of mHTT-induced neurodegeneration in vivo and screening the effects of putative therapeutic strategies. Although a detailed overview of the various genetic models of HD is outside of the scope of this review, a summary of the most common models is presented below, as they will be referred to in subsequent sections. Comprehensive review articles on this topic can be found elsewhere [127,128,129,130,131,132].

#### 1.2.1. Transgenic Truncated HD Mouse Models

The first murine HD genetic models to be developed, and arguably the most studied, are the R6 transgenic mouse lines (R6/1 and R6/2). The transgene used to engineer these transgenic lines contained HTT promoter sequences, exon 1 of HTT, and 262 base pairs of introns 1. These two lines vary in their transgene expression level, with R6/1 and R6/2 expressing it at approximately 31% and 75% of the level of the endogenous gene, respectively. The number of CAG repeats also varies between the two lines, with R6/1 and R6/2 mice expressing approximately 116 and 144 CAG repeats, respectively [133]. R6/1 mice typically begin to show signs of the disease between 15 and 21 weeks of age. Their symptoms progress slowly, beginning with clasping of the feet and progressing to tremor with the occasional occurrence of epileptic seizures. On the other hand, R6/2 mice, by far the most used, are characterized by an earlier onset of symptoms (at around 4 weeks of age) and faster progression of the disease [133,134]. Their lifespan is typically limited to 10–17 weeks, depending on the colony [133,135]. These mice are also known to display clasping of the feet, progressive resting tremor, and more frequent epileptic seizures when compared to the R6/1 line [133]. In addition to these symptoms, these transgenic mice also display marked brain and striatal atrophy and widespread NIIs of truncated mHTT [136]. However, striatal neuronal loss is limited [137,138].

N171-82Q mice constitute another truncated transgenic mouse model of HD. This model uses the mouse prion promoter to drive the expression of a cDNA coding a truncated fragment of the HTT gene that expresses the first 171 amino acids in the N-terminal region of the protein, along with 82 CAG repeats. Phenotypically, N171-82Q HD mice develop tremors, hypokinesia, and clasping of the limbs, albeit to a lesser degree than those seen in R6/2 mice. Unlike the R6/2 model, N171-82Q mice are indistinguishable from their wild-type littermates until approximately 2 months of age and survive for 6–11 months [139]. These mice also exhibit NIIs of mHTT fragments, progressive atrophy of multiple brain regions including the striatum, and more significant cell loss than that seen in the R6/2 model [139,140,141]. Both the R6 lines and the N171-82Q model are certainly useful and have contributed significantly to our current understanding of the neuropathological mechanisms triggered by mHTT fragments. However, since they only express a fragment of the full HTT gene, they are not helpful when studying the putative effects of therapies that may act on regions of the mutant protein that are not contained within the expressed fragment [142].

#### 1.2.2. Transgenic Full-Length HD Mouse Models

Given these limitations of the transgenic truncated models, alternate transgenic models that express the full-length mHTT gene have also been generated.

The two most used full-length transgenic models are the YAC128 and the BACHD lines. Both models have normal life expectancies and are slower in developing signs of disease. The YAC128 line uses a yeast artificial chromosome (YAC) to express the entire human HTT gene with 128 glutamines encoded by CAG and CAA repeats [143]. This line is similar to the human condition in that it demonstrates a biphasic pattern of activity, with early hyperkinesia followed by accelerated deterioration and late stage hypokinesia [144]. Motor and cognitive impairments are present from 3 months of age [143,144,145]. YAC128 mice exhibit atrophy of the striatum and cortex with concomitant neuronal loss [146]. In addition, increased nuclear huntingtin (but no NIIs) is present at 12 months of age. NIIs can be seen in striatal cells at 18 months of age [143,147,148].

The BACHD mouse model is a transgenic line that uses a bacterial artificial chromosome (BAC) to express the full-length human HTT gene with 97 CAG repeats under endogenous control. These animals have a similar phenotypic profile to that of the YAC128 model. They demonstrate progressive motor deficits from 2 months of age and synaptic dysfunction in the striatal MSN population. Unlike YAC128 mice, in BACHD mice, mHTT aggregates are primarily present in the cytoplasm rather than the nucleus [149,150].

Interestingly, unlike the human condition and the truncated models discussed above, full-length models exhibit increased weight gain rather than weight loss, making the study of the metabolic effects of HD challenging in these models [132]. It is also important to note that neither YAC128 nor BACHD mice exhibit germline or somatic instability [143,149], a feature that is closely related with the presence of mixed CAG/CAA repeats in these models and that contrasts with the human condition.

Of note, fully genetically humanized models have also been created by intercrossing BAC and YAC mice on an Hdh-/- (the mouse homolog of the human HTT gene) background to create Hu128/21 (YAC128/BAC21) and Hu97/18 (BACHD/YAC18) [151,152].

#### 1.2.3. Knock-in HD Mouse Models

In addition to transgenic mouse models, HD knock-in mouse models have also been created using various genetic manipulations. In general, knock-in models exhibit early development and slow disease progression [153]. In some models, an extended CAG sequence was inserted into the endogenous Hdh mouse gene [154], with the length of the CAG sequence varying significantly among models [132]. Alternatively, exon 1 of the endogenous Hdh gene was replaced with a mutated exon 1 of the human HTT carrying an expanded number of CAG repeats [153]. From a genetic point of view, knock-in animals are more accurate than transgenic ones since, in transgenic models, the mutated human gene is added into the mouse genome at random with the endogenous mouse Hdh gene still present.

One of the more recently generated knock-in models is the zQ175 line, which originated as a product of a germline CAG expansion in the Hdh140 knock-in line [155]. zQ175 animals exhibit motor symptoms from 4 to 8 weeks of age, hypoactivity from 4 months of age, and cognitive deficits from 12 months of age. Similar to the human condition and some of the transgenic models discussed above, these animals also experience weight loss. Neuropathologically, these animals show atrophy of both the striatum and neocortex along with NIIs within these brain regions [155,156]. Interestingly, the zQ175 line is the only HD mouse model in which the heterozygote phenotype is robust enough for use in therapeutic experimentation [132].

The HD genetic animal models described above are among the most commonly used in HD research. While none of these models perfectly mirror the human condition, they continue to constitute invaluable tools for pre-clinical studies, particularly in the context of screening of new therapeutic approaches for the treatment of HD-related symptoms.

## 2. Currently Available Treatments for HD

To date, no clinical trial has been successful in identifying disease-modifying treatments for HD [157,158,159,160,161], and therefore available therapeutic strategies remain primarily symptomatic. The current treatment regimens target motor, cognitive, and psychiatric symptom management and aim to improve the quality of life of those living with this disease. This section will discuss the currently available treatments for this neurodegenerative disorder.

### 2.1. Treatments to Manage Motor Symptoms

The predominant motor symptom in early HD is chorea, which, as explained above, is thought to result from the loss of GABAergic MSNs that connect the striatum to the external segment of the globus pallidus in the indirect pathway of the basal ganglia, resulting in the inhibition of the subthalamic nucleus and a subsequent hyperkinetic state. The pharmacotherapies currently available to modulate chorea aim to decrease DA neurotransmission with the net effect of reducing excessive movement (by decreasing DA-mediated activation/disinhibition of the basal ganglia) [162].

In addition to chorea, HD also encompasses other motor symptoms such as akinesia, dystonia, dysarthria, and dysphagia. However, the treatment options for these aspects of the HD motor phenotype are more limited [162].

#### 2.1.1. Hyperkinesia

##### Dopamine Modulators

Tetrabenazine: Tetrabenazine (TBZ), a vesicular monoamine transporter (VMAT2) inhibitor, was approved by the US Food and Drug Administration (FDA) for the treatment of chorea in HD in 2008 and is one of the two approved drugs for this use thus far [163]. It depletes monoamine (including DA) reserves by reversibly inhibiting VMAT2 and decreasing monoamine uptake into synaptic vesicles [164]. The use of TBZ for the treatment of chorea was supported by a randomized controlled trial (RCT) conducted by the Huntington Study Group (2006) [165], which found significant improvement in the Unified HD Rating Scale (UHDRS) in the TBZ-treated group compared to the placebo group. To further support the use of TBZ, Frank et al. (2008) [166] also conducted an RCT looking at the re-emergence of symptoms after TBZ discontinuation. The results found that discontinuation was positive for re-emergent chorea, although a withdrawal effect could not be excluded. Of note, some adverse effects are seen with TBZ, including depression and parkinsonism, which are thought to result from the concomitant depletion of other monoamines, such as serotonin and norepinephrine [162]. These side effects are not to be taken lightly, given that HD patients are already at higher risk of depression, anxiety, and suicide when compared to the general population [3,167].

Deutetrabenazine: Deutetrabenazine is the second FDA-approved drug for the treatment of HD chorea [163]. As a deuterated form of TBZ, it has an improved risk–benefit profile when compared to TBZ, with a longer half-life, lower peak concentrations, and an unchanged pharmacological target effect, which together result in a lower dosage and frequency requirement as well as an overall lower risk of adverse side effects, including depression [168]. Though there has been no direct comparison to TBZ, the Huntington Study Group et al. (2016) [168] conducted an RCT comparing the effects of deutetrabenazine vs. control and showed that the deutetrabenazine-treated group had statistically significant improvements in the UHDRS chorea score and the UHDRS total motor score, as well as in several secondary endpoints, including the patients’ global impression of change, the clinical impression of change, and changes in physical function with no increase in depression, or sedation. However, it appears that this drug may increase the risk for suicidality [168].

##### Dopamine Antagonists

Neuroleptics: Neuroleptics act by blocking DA neurotransmission, and their potential benefits include treatment of chorea, agitation, and psychosis. Though the evidence-based guidelines for the pharmacological treatment of chorea from the American Academy of Neurology stated that no sufficient data are currently available to support the use of DA antagonists [164], according to the international group of HD experts, antipsychotic drugs were the drugs of choice in patients presenting with both chorea and psychiatric symptoms such as depression [169]. Due to each antipsychotic agent having varying degrees of affinity for the dopamine receptor, their efficacy in treating chorea also varies [162]. In patients with severe chorea, first-generation neuroleptic medications such as haloperidol may be useful; however, second-generation neuroleptic medications are preferred in those with moderate chorea due to a lower risk of extrapyramidal symptoms such as the development of tardive dyskinesia [170,171].

Typical neuroleptics: A review by Bonelli and Wenning (2006) [172] demonstrated a possible benefit for the treatment of HD chorea using a combination of haloperidol and fluphenazine. Though these first-generation antipsychotic drugs pose a high risk of adverse side effects such as the development of extrapyramidal symptoms including tardive dyskinesia, parkinsonism, dystonia, akathisia, and hypotension and thus are deemed inferior when compared with the newer second-generation antipsychotic drugs with less risk of adverse effects, many HD experts still indicate haloperidol as the first choice of antipsychotic drug in their practice due to its low cost [169].

Atypical neuroleptics: Olanzapine is an atypical neuroleptic used for various psychiatric conditions. In a review by Bonelli and Wenning (2006) [172], olanzapine was considered possibly efficacious in the treatment of HD chorea. An open-label study by Bonelli et al. (2002) [173] showed a positive response on the motor sub-scores of the UHDRS. However, a more robust randomized trial is warranted to establish the clinical value of olanzapine in HD. Aripiprazole is another atypical neuroleptic that was reported to be efficacious in the treatment of chorea in a case series of three patients, as well as a crossover trial of six patients, as compared to TBZ [174,175]. Finally, risperidone and quetiapine have also been reported to be beneficial in treating HD chorea in case reports, but no robust trials have yet been performed [171,173,176,177,178,179]. Similar to other antipsychotics, their side effects include sedation and parkinsonism due to DA antagonism.

##### Anti-Glutamatergic Drugs

Amantadine: Amantadine is an N-methyl-D-aspartate (NMDA) receptor antagonist, thought to counteract excessive glutamate neurotransmission in the basal ganglia by reducing NMDA receptor-mediated excitotoxicity and the consequent degeneration of striatal neurons in the HD brain (see Section 1.1.1). In addition, amantadine also has a poorly understood effect on DAergic synapses [162]. Amantadine is currently used as an effective treatment of dyskinesias in Parkinson’s disease, and thus it was thought that it might also be beneficial in the treatment of HD chorea. However, several studies only showed a modest beneficial effect of amantadine on HD chorea through improvements in the UHDRS [180,181,182]. Furthermore, due to the high doses of amantadine required to achieve symptom benefit, patients also experienced many adverse side effects such as hallucinations, forgetfulness, agitation, exacerbation of morbid thoughts, and sleepiness [182,183].

Riluzole: Similar to amantadine, riluzole has anti-glutamatergic properties, and several RCT studies have demonstrated the efficacy of different doses of this drug [184,185]. It was concluded that riluzole has an effect on reducing HD symptoms in a dose-dependent manner, with higher doses having more efficacy at 8 weeks. However, no improvement in functional capacity was noted [184]. Its adverse effects included reversible elevation in liver enzymes and increased risk of suicide [184,185].

#### 2.1.2. Hypokinesia and Rigidity

Unfortunately, treatment of chorea with the drugs mentioned above can unmask or exacerbate bradykinesia and rigidity. Treatment with DA agonists or DA replacement (levodopa) has been tried, but the efficacy of these agents has been limited to case series and reports [172,173,186]. Therefore, the usefulness of DA agonists and levodopa to treat symptoms of hypokinesia and rigidity in HD is limited, and these drugs are used on a case-by-case basis by experts in movement disorders and/or HD.

### 2.2. Treatments to Manage Non-Motor Symptoms

Although HD is widely recognized as a motor disorder, with chorea as the most recognizable motor impairment, non-motor symptoms may have a greater impact on the quality of life of HD patients and their families [162], particularly during the early stages of disease progression. In agreement, a recent study with 155 participants with manifest and prodromal HD identified that the three types of symptoms with a higher impact on participants were: difficulty thinking, impaired sleep or daytime sleepiness, and emotional issues [187]. Therefore, it is important to recognize and address these aspects of the disorder, to improve the overall quality of life of those living with this debilitating disease.

#### 2.2.1. Treatment of Cognitive Impairment

Cognitive symptoms can manifest decades before the diagnosis of HD and the onset of motor symptoms. As stated above, the cognitive deficits seen in HD are thought to be primarily subcortical and include decreased processing speed, poor attention, poor problem-solving abilities, and poor memory retrieval [188]. These qualities are difficult to assess with the traditional screening measures for cognitive dysfunction, such as the Folstein Mini-Mental State Examination (MMSE). This, in conjunction with the patients’ poor insight into their own cognitive abilities due to dysfunction of the frontal-striatal connections, is known to result in safety concerns and added caregiver stress [189]. Unfortunately, there is no known effective therapy for dementia associated with HD. Clinical data for cholinesterase inhibitors typically used for non-HD cognitive impairment such as donepezil, rivastigmine, and galantamine do not show a clear benefit [190,191,192,193].

Memantine, a non-competitive moderate-affinity NMDA receptor antagonist that stabilizes glutamatergic tone, was postulated to be beneficial in HD patients with cognitive impairment, given that glutamate-mediated excitotoxicity is thought to mediate, at least in part, neurodegeneration, and striatal neuronal death in the HD brain (see Section 1.1.1) [162,194]. However, despite its use in Alzheimer’s dementia, its use in HD is currently understudied, with only one small open-label report suggesting a potential neuroprotective effect with long-term use [195].

#### 2.2.2. Depression

Depression is the most common psychiatric symptom reported by individuals living with HD [3]. Its prevalence is greatest around the time patients begin to lose their independence [196,197]. The pathophysiology of this symptom is likely related to the degeneration of striatal circuits involving the frontal lobe as well as the ventral anterior and the medial dorsal nuclei of the thalamus [198]. Though there is insufficient evidence to guide pharmacotherapy of depression in HD as shown by a systematic review [199], patients commonly experience benefits from treatment of their depression with selective serotonin reuptake inhibitors (SSRIs) [162]. Though older agents such as tricyclic antidepressants may also be beneficial, due to their increased risk of adverse side effects affecting cognition, these are often considered second line [200]. Depression refractory to SSRI treatment can be treated with atypical antipsychotics such as olanzapine, risperidone, aripiprazole, and clozapine [174,175,201,202,203,204].

#### 2.2.3. Other Behavioral Symptoms

Patients with HD experience other behavioral and psychiatric symptoms such as apathy, irritability, obsessive-compulsive disorder, and psychosis. Apathy is the most prevalent in the advanced stages of the disease and has been reported in up to 50% of large cohort studies [196,197,198]. Unfortunately, there are no known treatments for this symptom; however, patients may benefit from reducing the dose of their anti-chorea medication if chorea is less of an issue at this stage, as these medications can contribute to apathy [162]. Irritability is prevalent in 38–73% of patients [199], and HD experts recommend SSRIs in milder cases and atypical antipsychotics or mood-stabilizing antiepileptics in more severe cases [205]. Obsessive-compulsive disorder can be seen in 10–50% of patients with HD [196]. There is no evidence-based treatment for this symptom in HD patients, but HD experts recommend SSRIs, clomipramine, antipsychotics, and mood-stabilizing antiepileptic drugs as possible options [206,207]. Psychosis is uncommon in HD but has been reported in 3–11% of patients, with symptoms worsening with disease progression [196]. Atypical antipsychotics such as olanzapine and risperidone are the recommended pharmacologic treatments, and patients should be closely monitored for potential adverse side effects such as hypokinesia [176,177,208,209].

## 3. Clinical Trials

**Figure 1:**
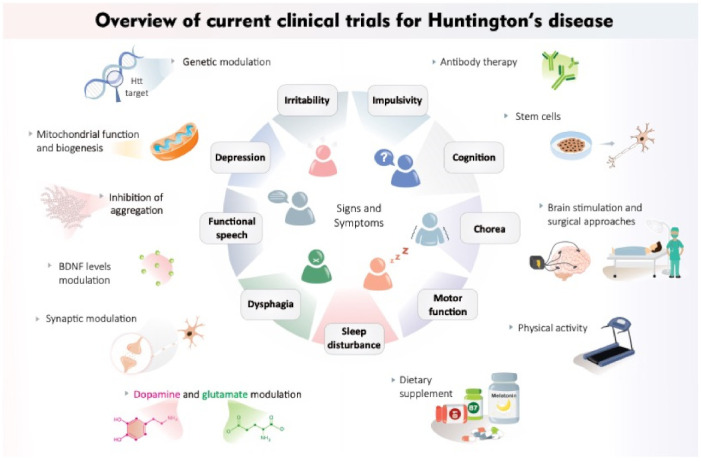
Overview of current clinical trials for Huntington’s disease.

Since the currently available treatments for HD are primarily focused on symptom management and no cure is yet available for this devastating disorder, the search for new and effective HD therapies remains a priority. Within this scenario, the potential efficacy of numerous treatment modalities for HD is currently being tested through clinical trials. Table 1 lists all clinical trials currently active and/or recruiting participants and listed on Clinicaltrials.gov. A brief summary of each intervention and its potential mechanism of action is listed below (see also Figure 1).

### 3.1. Dopaminergic Modulation

Tetrabenazine: As discussed above (see Section 2.1.1), TBZ is a VMAT2 inhibitor that depletes DA reserves and is used to manage chorea in HD [163]. The current clinical trial (NCT02509793) aims to further analyze its effect on behavioral symptoms (including depression) that result from TBZ-induced serotonin depletion [162] and assess any potential beneficial effects on impulsivity.

Deutetrabenazine: Deutetrabenazine is a deuterated form of TBZ, with the substitution of deuterium for hydrogen at key positions in the TBZ molecule allowing for a longer half-life and less frequent daily dosing. There are currently two clinical trials with deutetrabenazine. NCT04301726 is testing the efficacy to control symptoms of dysphagia associated with HD, whereas NCT04713982 is investigating the impact of deutetrabenazine on functional speech and gait dynamics.

Valbenazine: Valbenazine is the pro-drug of the alpha isomer of TBZ, making it the most selective enantiomer for VMAT2 [210]. Valbenazine is currently FDA-approved for usage in the treatment of tardive dyskinesia [210]. Given its similar chemical structure to TBZ and the efficacy of TBZ in treating HD chorea, this clinical trial (NCT04102579) aims to evaluate the efficacy, safety, and tolerability of valbenazine in treating chorea in HD patients.

Risperidone: Risperidone acts as a high-affinity DA D2 receptor antagonist and serotonin 5-HT2A receptor antagonist [211]. The current clinical trial (NCT04201834) aims to assess the safety and benefits of risperidone in the treatment of chorea in HD patients.

### 3.2. Glutamatergic Modulation

Dextromethorphan/Quinidine (DM/Q): Dextromethorphan is an NMDA antagonist that is rapidly metabolized to dextrorphan by cytochrome P450 2D6 (CYP2D6) [212]. To increase the plasma bioavailability of dextromethorphan, it is administered in combination with quinidine, a CYP2D6 inhibitor [212]. Due to dextromethorphan’s antagonistic properties at the NMDA receptor and, therefore, its potential ability to decrease excitotoxicity in the HD brain, this clinical trial (NCT03854019) is investigating the putative therapeutic effects of this combination of drugs on HD chorea.

### 3.3. Synaptic Modulation

Neflamapimod: This is a small molecule that can penetrate the brain and inhibit the enzyme p38-alpha, which is typically involved in regulating inflammation and, if chronically activated, can negatively affect nerve cell communication due to excessive inflammation [213,214]. Several studies using mouse models have shown that neflamapimod could be beneficial in treating neurodegenerative diseases such as HD by reversing synaptic dysfunction [213,214]. Given this, the current clinical trial (NCT03980938) aims to determine whether neflamapimod can reverse hippocampal dysfunction in patients with early-stage HD.

### 3.4. Modulation of BDNF Levels

Pridopidine: Pridopidine is a first-in-class sigma-1 receptor (S1R) agonist and DA D2 receptor antagonist. S1R plays a key role in neuroprotection through the increased production of BDNF levels. The current clinical trial (NCT04556656; PROOF-HD Study) is a phase 3 randomized, placebo-controlled study that aims to investigate the functional benefit of this drug in HD.

### 3.5. Mitochondrial Function and Biogenesis

Fenofibrate: Fenofibrate is a peroxisome proliferator-activated receptor (PPAR) agonist that may induce activation of PGC-1α and aid in mitochondrial biogenesis [215]. Since mitochondrial biogenesis is thought to be impaired in HD due to faulty expression of PGC-1α ([215]; see Section 1.1.3), this clinical trial (NCT03515213) is aimed to investigate whether pharmacological activation of PGC-1α will be of benefit in treating HD.

Triheptanoin: Triheptanoin is a C7 fatty acid that produces acetyl-CoA and propionyl-CoA upon being metabolized by the liver [216]. Reduced concentrations of branched-chain amino acids have been found in patients with HD, and this may reflect underlying alterations in mitochondrial oxidation, in order to continue to provide acetyl-CoA and succinyl-CoA, which fuel the Krebs cycle [216]. Triheptanoin may provide substrates to the Krebs cycle, thus increasing ATP availability and correcting the bioenergetic profile in the HD brain [216]. The current clinical trial (NCT02453061) aims to test the efficacy of triheptanoin on the motor and neuropsychological domains, and on the overall patient quality of life. Caudate volume will be measured using volumetric magnetic resonance imaging and brain energy metabolism.

Metformin: Metformin is an AMP-activated protein kinase (AMPK) activator commonly used to treat type 2 diabetes. Metformin was shown to be protective in polyQ-expressing C. elegans and in vitro HD models [217,218]. In addition, metformin treatment increased the lifespan of male R6/2 HD transgenic mice [219] and alleviated the neuropsychiatric and motor phenotypes of zQ175 mice [218]. Furthermore, a statistical analysis of the Enroll-HD database showed that metformin intake is associated with better cognitive function in HD patients [220]. Given these data, the current clinical trial (NCT04826692) aims to assess the efficacy and safety of metformin treatment against cognitive decline in HD.

### 3.6. Aggregate Inhibition

Nilotinib: Phosphorylation and subsequent activation of Abelson (Abl), a tyrosine kinase involved in a variety of functions including apoptosis, have been detected in neurodegenerative diseases [221]. In Parkinson’s disease, Abl levels, as well as Lewy bodies (inclusions primarily containing aggregated alpha-synuclein), are elevated in nigrostriatal regions [222,223], and the inhibition of Abl has resulted in increased survival of dopaminergic neurons in Parkinson’s disease models [223]. Nilotinib (Tasigna™) is an Abl inhibitor and autophagy modulator [224,225,226,227], known to interact with Beclin1 through a Bcl-2 homology 3 (BH3) domain [228]. Given the relationship between Abl and neurodegeneration, Abl inhibition with nilotinib is thought to decrease the accumulation of alpha-synuclein [229,230]. Notably, overexpression of alpha-synuclein in mouse models of HD enhances the onset of tremors and weight loss [231]. However, a recent study showed no changes in autophagy and aggregation levels and no behavioral alterations in R6/2 mice treated with nilotinib (Tasigna™) [228]. Nevertheless, a current clinical trial (NCT03764215) aims to evaluate whether nilotinib can have beneficial effects in patients with HD.

### 3.7. Antibody Therapy

ANX005 is a monoclonal antibody designed to inhibit C1q, the initiating molecule of the complement cascade of the innate immune system that functions primarily as a first-line host defense against infections [232,233]. The safety and tolerability of ANX005 are currently being evaluated in subjects with manifest HD, or those at risk of developing this disorder (NCT04514367).

### 3.8. Genetic Manipulations

IONIS-HTT: This is a non-allele-specific antisense oligonucleotide (ASO) that inhibits mRNA and reduces both mHTT and wild-type HTT expression [234]. A randomized controlled trial in phases 1–2a involving intrathecal administration of IONIS-HTT vs. placebo in adults with early HD was conducted by Tabrizi et al. (2019) [235]. This study showed a dose-dependent reduction in mHTT in the cerebrospinal fluid (CSF) [235]. Two studies (NCT03842969; NCT04000594) are currently investigating the efficacy and safety of this drug.

AMT-130: This is a microRNA targeting human HTT, delivered via adeno-associated viral vector serotype 5 (AAV5-miHTT), which has been shown to efficiently lower normal and mutant HTT levels both in vitro and in vivo (i.e., rodent models) [236,237,238,239,240,241]. There is one active proof-of-concept (POC) trial (NCT04120493) currently recruiting adults with early manifest HD to test the safety of intrastriatal delivery of AMT-130.

Although genetic approaches seem the most promising because of the genetic nature of HD, this year, at least five clinical trials (NCT03761849, NCT03225833, NCT04617847, NCT03225846, NCT04617860) have been stopped due to a lack of efficacy, as reported on clinicaltrials.gov. The experimental drug RO7234292 (NCT03761849) was already in phase 3 when studies were stopped in March 2021. The decision of discontinuation of what appeared to be a promising drug was based on recommendations from the independent Data Monitoring Committee (iDMC) [242].

### 3.9. Dietary Supplementation

Melatonin Supplementation: Melatonin is a methoxyindole synthesized and secreted by the pineal gland at night under normal light/dark conditions [243]. A current clinical trial (NCT04421339) has been designed to study the potential effects of melatonin supplementation on HD patients afflicted with HD-related sleep disturbance.

Combined Thiamine and Biotin Supplementation: Biotin–thiamine-responsive basal ganglia disease (BTBGD) is a movement disorder characterized by recurrent subacute encephalopathy manifested as confusion, seizures, ataxia, dystonia, supranuclear facial palsy, external ophthalmoplegia, and/or dysphagia which, if left untreated, can eventually lead to coma and death. Immediate supplementation with biotin and thiamine early in the course of the disease results in partial or complete improvement when the disease presents during childhood or adulthood [244]. Given this, a current clinical trial (NCT04478734) aims to evaluate the safety and tolerability of combined oral thiamine with biotin therapy in patients with mild to moderate HD.

### 3.10. Combined Pharmacological Therapies

There is currently one clinical trial (NCT04071639) testing the efficacy of a combination of drugs (deutetrabenazine, risperidone, Zoloft, and idebenone) at providing symptomatic relief at different stages of disease progression. As outlined above, deutetrabenazine and risperidone are dopaminergic modulators, whereas idebenone is a synthetic coenzyme Q10 mimetic with antioxidant properties. Of note, a previous 1-year, double-blind, parallel group clinical trial tested the effects of idebenone treatment in HD patients. However, no significant differences between groups on the primary outcome measures of the Huntington’s Disease Activities of Daily Living Scale (ADL—an index of functional status) and the Quantified Neurologic Examination (QNE) were observed [245]. Nevertheless, it might be that combining idebenone with deutetrabenazine and risperidone will prove more beneficial.

### 3.11. Stem Cell Therapies

Cellavita: Stem cell therapies with neural progenitor cells (NPCs) derived from induced pluripotent stem cells (iPSCs) have been noted to hold considerable potential for the treatment of neurodegenerative diseases, including HD [246]. In animal studies, human iPSC-derived neural progenitor cells were found to engraft into the brain and differentiate into normal neuronal cells, promoting behavioral and motor recovery [247]. Three clinical trials are currently active to assess the dose (NCT03252535), safety, and efficacy (NCT02728115; NCT04219241) of intravenous delivery of stem cells in human HD patients.

### 3.12. Deep Brain Stimulation (DBS)

DBS has been used for a long period of time for various neurological and psychiatric disorders, with varying degrees of success [248]. Indeed, DBS has been reported to improve symptoms such as tremor, dyskinesia, chorea, and dystonia [249,250,251]; however, its mechanism of action is yet not completely understood. It is thought that activation of the stimulated area, with concomitant inhibition of the surrounding area, increases the frequency of action potentials in the area of interest [252]. The globus pallidus is the preferred brain region targeted for DBS in HD. Indeed, DBS in this region has been shown to reduce levodopa-induced dyskinesias in patients with Parkinson’s disease [253,254]. Moreover, several studies have found that stimulation of the globus pallidus can significantly improve chorea [253,254,255,256,257,258,259,260,261,262,263,264]. The current three clinical trials (NCT02535884; NCT04244513; NCT04429230) aim to further investigate the efficacy of this strategy.

### 3.13. Physical Activity

Improvements in levels of fitness, motor function, and quality of life have been demonstrated in some clinical studies using short-term exercise interventions in HD patients [265,266,267,268,269,270]. Unfortunately, long-term studies on the effects of exercise in HD patients are logistically challenging [271]. The current clinical trial (NCT03344601) aims to use a “trials within cohorts” (TWiCs) design for efficient patient recruitment, and prospective outcomes will be routinely collected to facilitate data collection [271]. Moreover, an additional clinical trial involving a 4-week exercise program (NCT04917133) aims to test the effects of adapted physical activity (APA) during a rehabilitation stay on motor, cognitive, and psycho-behavioral abilities in mid-stage HD patients.

## 4. Pre-Clinical Experimental Therapeutic Approaches

Despite our increasing understanding of the mechanisms underlying HD and the fact that its genetic cause has been known for almost 30 years, the current treatment options for this disorder are minimal (see Section 2). In addition, although current clinical trials may lead to the identification or refinement of treatments that are likely to improve the quality of life of those living with this debilitating disease (see Section 3), there is still no cure in sight. Given this, significant efforts continue to be invested at the pre-clinical level, with numerous studies currently testing the potential beneficial effects of novel therapeutic approaches. Although a comprehensive overview of all pre-clinical studies conducted in HD models is outside of the scope of the present review, the following sub-sections will focus on some of the therapeutic strategies that are thought to have potential as disease-modifying approaches. These include increasing neurotrophic support, autophagy modulation, epigenetic and genetic manipulations, and the use of stem cells and nanocarriers.

### 4.1. Neurotrophic Factors

#### 4.1.1. Brain-Derived Neurotropic Factor (BDNF)

The strategies for restoring BDNF levels can vary significantly [272]. Although it is possible to increase BDNF levels with dietary interventions [109,273] or through environmental enrichment [274,275] and physical activity [113,276,277], here, we focus on the direct up-regulation of this neurotrophin through protein infusion, genetic approaches, and pharmacological strategies.

Up-Regulation of BDNF Levels by Protein Infusion and Genetic Approaches:

Various studies have assessed the effects of up-regulating BDNF protein levels or gene expression in different animal models of HD. In most cases, delivery of BDNF by protein infusion through the implantation of BDNF mini-pumps [278], intrastriatal injection of adenovirus expressing BDNF [279,280], or implantation of cells expressing BDNF [281,282,283,284,285] was shown to induce some degree of striatal neuroprotection and motor improvement [126,272,286]. Despite these promising results, the use of mini-pumps is associated with some disadvantages that should be considered when extrapolating these results to the clinic. First, the degree of invasiveness of this procedure might not be practical or even feasible in the clinical setting. Second, this administration route will likely generate a steep concentration gradient, originating from the infusion point, leading to alterations in the infused tissue and the development of adverse side effects, such as edema [287]. Thus, other approaches to efficiently deliver optimum doses of BDNF to the brain are currently considered more feasible candidates for clinical trials in HD.

An attractive alternative approach is gene therapy using viral vectors containing the *BDNF* gene, which allows for the constant and local production of the protein by the desired neuronal population. Moreover, gene therapy also overcomes the problem of protein instability that may result from the long-term storage of BDNF inside the pumps. Given this rationale, Cho and colleagues (2007) [279] tested the potential beneficial effects of administering a combination of adenoviral BDNF (AdBDNF) and adenoviral noggin (AdNoggin) via intraventricular injections to 4- and 6-week-old R6/2 mice. A combination of both AdBDNF and AdNoggin was shown to increase the recruitment of new neurons from the subventricular zone into the striatum of R6/2 mice. Furthermore, these newly generated neurons were shown to mature into dopamine- and cAMP-regulated phosphoprotein of molecular weight 32 kDa (DARPP-32)-positive GABAergic medium-sized spiny neurons that expressed either enkephalin or substance P and extended fibers to the globus pallidus. Importantly, AdBDNF/AdNoggin treatment also improved rotarod performance and open field activity and increased the survival of R6/2 mice by 16.8% [279]. In a different study, Arregui et al. (2011) [288] induced adenovirus-mediated expression of BDNF in astrocyte cells. Of note, delivery of this adenoviral construct into the striatum of R6/2 HD mice resulted in delayed onset of their motor phenotype, as assessed by paw clasping, an open field test, horizontal and vertical movement, wire hang endurance, and rotarod [288].

Nevertheless, although *BDNF* gene therapy appears as a promising candidate for clinical trials, there are still a few drawbacks that should be overcome before this strategy can be used in the clinical setting. The first problem is the regulation of the amount of BDNF produced locally, as an excess of total BDNF can potentially be deleterious, namely, if the mechanisms of BDNF processing are compromised, leading to the accumulation of increased amounts of pro-BDNF, which is known to have pro-apoptotic functions [289]. Secondly, transduction is often associated with inflammation, which may also result from vector toxicity and prevent long-term therapy. Another major problem is the risk of accidental insertional mutagenesis by viral vectors and subsequent tumor formation [290]. To overcome these problems, new adeno-associated viral vectors lacking pathogenicity and immunogenicity as well as nontoxic viral systems [291,292] are currently being investigated.

Another alternative to increasing BDNF levels locally is by striatal transplantation of cells engineered to stably express BDNF. This approach has been tested in animal models of HD, using either immortalized rat fibroblasts genetically engineered to secrete BDNF or human embryonic neural progenitors [281,282,283,284,285]. Overall, these strategies improved motor performance and reduced striatal neuropathology (for review, see [126]). Since xenogenic grafts can be rejected by the host and immortalized cells can cause tumor growth, research is currently focused on developing stable, non-tumorigenic human neural stem cell lines that can express BDNF (for review, see [293,294]).

Up-Regulation of BDNF Levels by Pharmacological Strategies:

Several studies have tested the ability of different pharmacological interventions to increase endogenous BDNF levels using in vivo models of HD. These strategies would circumvent the problems related to the use of invasive methods of BDNF delivery while allowing for the correct dosage and stability of this neurotrophin.

Within this scenario, the non-competitive inhibitor of ionotropic glutamate NMDA receptors riluzole has been shown to increase BDNF expression [122] and ameliorate HD symptoms in HD transgenic mouse models [295,296] and human HD patients [122]. On the other hand, cystamine, an inhibitor of transglutaminase, is a neuroprotective drug that inhibits caspase-3 activation [297] and increases the levels of the antioxidant’s glutathione and L-cysteine [297,298]. Furthermore, cystamine has been shown to increase the transport and release of BDNF from brain neurons [299]. In agreement, cystamine and its Food and Drug Administration (FDA)-approved derivative cysteamine were shown to be effective in increasing BDNF levels and consequently inducing neuroprotective effects in HD mouse models and in up-regulating serum BDNF levels in mouse and primate models of HD [299].

BDNF can also be regulated by activation of the serotonergic system (for review, see [300]), and administration of selective serotonin reuptake inhibitors (SSRIs) was shown to increase the levels of BDNF in the hippocampus [301,302,303,304]. Thus, SSRIs might be used to modulate the levels of BDNF in the HD brain. In agreement, various studies have shown that treatment with different SSRIs can have beneficial effects on disease progression in HD transgenic models. Paroxetine was found to attenuate motor dysfunction and body weight loss while increasing the lifespan of HD N171-82Q mice [305]. Fluoxetine treatment rescued the deficits in hippocampal neurogenesis and improved hippocampal-dependent cognitive function in R6/1 mice. However, no effects on the locomotor deficits and the loss of body weight were observed [306]. In a more recent study, daily administration of sertraline to R6/2 mice (starting at 6 weeks of age) did not affect the loss of body weight or the number of NIIs. However, sertraline-treated mice survived longer, performed better on the rotarod test, and showed reduced striatal atrophy. Importantly, sertraline also ameliorated the impairment in hippocampal neurogenesis and attenuated the depletion of BDNF in the striatum and hippocampus of these HD mice [114]. Similar results were also found in the N171-82Q HD transgenic mouse model [307]. Indeed, although not affecting body weight or the number of NIIs in these mice, sertraline treatment was shown to improve their brain atrophy, motor performance, and survival [307]. Furthermore, sertraline-treated N171-82Q HD mice also had normalized cortical BDNF and striatal serotonin levels as well as enhanced adult neurogenesis, which might mediate, at least in part, the beneficial effects of sertraline in these mice [307]. Notably, the serum and brain levels of sertraline that were shown to be beneficial in these transgenic mice are comparable to the levels achieved in humans treated with this antidepressant [308], making sertraline an interesting candidate for HD clinical trials. More recently, Cong et al. (2015) [309] demonstrated that treatment with the commonly used tricyclic antidepressant (TCA) amitriptyline (starting at 2 months of age and continuing for 6 weeks) also enhanced BDNF striatal levels and reduced mHTT aggregation in N171-82Q mice. Such results were correlated with a positive effect on motor performance on the beam test and rotarod.

Laquinimod, a new quinoline-3-carboxamide immunomodulator currently being tested for some neurodegenerative diseases such as multiple sclerosis, is another compound that has also been shown to increase BDNF levels and positively impact motor function in both R6/2 [310] and YAC128 [311] HD mice. Glatiramer acetate (GA) is another immunomodulator that has been approved for the treatment of multiple sclerosis [312,313]. Though its mechanism of action is not fully elucidated, it is thought that this drug can increase BDNF and downregulate the levels of proinflammatory cytokines. A recent study tested the effects of GA on CAG140 KI mice (0.625 mg/mouse, three times per week beginning at 3 months until 1 year of age) and N171-82Q mice (1 mg/mouse, five times per week, from 8 to 20 weeks of life), and significant motor improvements were seen in both mouse models as assessed through rotarod performance, climbing, and open field activity test [312]. Additionally, in vitro and in vivo studies demonstrated that GA could increase BDNF levels in astrocytes of both R6/2 and YAC128 HD mice and prolong the lifespan of R6/2 mice [313].

Various studies have also suggested that targeting BDNF signaling might also improve non-motor deficits in HD mouse models. Anglada-Huguet et al. (2016) [314] treated 10-week-old R6/1 HD mice with misoprostol, a prostaglandin EP2 receptor agonist. Misoprostol was able to reverse deficits in BDNF levels, increase dendritic branching in a BDNF-dependent manner in hippocampal neurons, and reduce the number of mHTT NIIs in the hippocampal dentate gyrus. In addition, misoprostol improved long-term memory as assessed with the novel object recognition test and the T-maze spontaneous alternation test [314]. In a different study, Da Fonseca et al. (2018) [315] demonstrated the effects of intranasal administration of human recombinant BDNF for 15 days on the occurrence of depressive-like and anhedonic behaviors in YAC128 HD mice. Although BDNF treatment did not alter striatal or hippocampal BDNF levels, behavioral improvements were observed, as assessed with the open field test, splash test, sucrose preference test, tail suspension test, and forced swim test [315]. Similar studies have used different intranasal agents that are thought to exert some of their positive effects on HD pathology by increasing the release of BDNF. Cabezas-Llobet et al. (2018) [316] used intranasal pituitary adenylate cyclase-activating polypeptide 38 (PACAP38) to treat R6/1 mice for 7 days at the onset of cognitive impairment. PACAP38-treated mice showed cognitive improvements thought to be, at least in part, attributed to increased BDNF expression [317]. Similarly, a study completed by Fatoba et al. (2018) [318] demonstrated that intranasal administration of neuropeptide Y resulted in increased BDNF expression and decreased aggregation of mHTT in R6/2 mice [318].

#### 4.1.2. Glial Cell Line-Derived Neurotropic Factor (GDNF)

GDNF has been identified as a crucial trophic factor in dopaminergic neuronal development and for the neuroprotection of the dopaminergic system [316,319,320,321].

In an initial study using a QA lesion model, intracerebroventricular GDNF 30 min before QA administration prevented neuronal degeneration [322]. Although these were promising findings, because GDNF was administered before striatal lesioning by quinolinic acid, it was uncertain whether GDNF could also be neuroprotective once the neurodegeneration was already established. Nevertheless, it is reasonable to speculate that GDNF could still provide symptomatic relief by preventing further deterioration of the striatum. In a subsequent study, the potential protective effects of enhancing GDNF expression through an adeno-associated virus carrying the *GDNF* gene (AAV-GDNF) were also assessed. It was found that when AAV-GDNF was administered 3 weeks before quinolinic acid, it protected against loss of striatal neurons [280]. Similarly, bilateral striatal injections of AAV-GDNF were also shown to be protective in a 3-nitroproprionic acid (3-NP, an inhibitor of mitochondrial respiratory chain complex II) rat model of HD [323]. When administered two weeks before 3-NP infusion, AAV-GDNF was able to reverse 3-NP-induced motor deficits and normalize gait abnormalities in all AAV-GDNF- and 3-NP-treated animals [324].

Following these studies in toxin-induced lesion HD models, McBride and colleagues (2006) [324] also tested the effects of AAV-GDNF treatment on N171-82Q transgenic HD mice. Treatment of 5-week-old pre-symptomatic N171-82Q mice with AAV-GDNF resulted in motor improvements (as assessed with the rotarod test and hind limb clasping). Indeed, AAV-GDNF-treated N171-82Q mice showed increased motor performance when compared with their N171-82Q counterparts treated with a control viral vector (AAV-green fluorescent protein (GFP)) from 10 to 16 weeks of age, and AAV-GDNF-treated N171-82Q mice performed similarly to WT mice up until the last three weeks of testing. Moreover, AAV-GDNF N171-82Q mice showed an increased number of striatal neurons (which were also larger) and a reduction in the number of striatal inclusions when compared with AAV-GFP-treated N171-82Q mice [324]. However, in a different study, intrastriatal administration of a GDNF-containing lentiviral vector was unable to alleviate behavioral or neurological effects in R6/2 mice when administered at 5 weeks of age, when these animals started to display some of the symptoms of the disease [325]. Thus, it appears that GDNF would likely be best utilized in the early, pre-symptomatic stages of HD. Furthermore, differences in the viral vectors used in the different studies may account, at least in part, for these discrepancies, and further optimization of the protocols of viral-mediated gene transfection is thus warranted.

#### 4.1.3. Other Neurotrophic Factors

The potential neuroprotective effects of other neurotrophic factors such as ciliary neurotrophic factor (CNTF), vascular endothelial growth factor (VEGF), and insulin-like growth factor −1 (IGF-1) have also been studied in HD animal models, and a brief overview of these studies is presented below.

CNTF is a pluripotent neurotrophic factor that plays a role in neuronal differentiation and survival, and its potential therapeutic properties have been evaluated in different neurodegenerative disorders, such as amyotrophic lateral sclerosis (ALS) [326,327]. In agreement, [328] demonstrated that CNTF could protect striatal neurons and improve striatal neuronal survival in quinolinic acid-lesioned rats when delivered through osmotic pumps into the striatum. In a subsequent study, Emerich et al. (1997) [329] assessed the effects of human CNTF expression in a QA non-human primate HD model. Cynomolgus primates received unilateral implants of polymer-encapsulated baby hamster kidney fibroblasts genetically modified to produce human CNTF one week before lesioning of the striatum by QA administration. CNTF-treated primates showed substantial preservation (approximately 64%) of striatal cells and reduced atrophy of the motor cortex as compared to controls.

De Almeida et al. (2001) [330] demonstrated that intrastriatal administration of a CNTF-containing lentiviral vector to rats that were lesioned with QA three weeks later significantly reduced striatal damage [330]. However, despite these promising results obtained in various HD lesion models, CNTF is also associated with disruptive side effects that limit its use in the clinic. Indeed, muscle pain, cramping, hyperalgesia, profound weight loss, and other severe dose-dependent side effects have been reported in the literature [331,332]. Nevertheless, this growth factor was shown to be useful in retinal degeneration, and further research is currently underway to devise strategies to surpass its side effects [327].

IGF-1 is a hormone primarily produced by the liver that is similar to insulin, playing an essential role in maintaining blood sugar levels. IGF-1 primarily binds its own tyrosine kinase receptor, but due to its similarity with insulin, it can also bind the insulin receptor with weaker affinity [333]. The role of IGF-1 in HD has been primarily studied in the context of the metabolic dysfunction (e.g., increased blood glucose levels) that is often manifested with this disease [334]. Nevertheless, Lopes et al. (2014) [335] showed that intranasal IGF-1 administration to YAC128 HD transgenic mice significantly improved their performance both in the rotarod and open field tests. However, no metabolic benefits were observed with this treatment regimen [336].

VEGF has classically been known to promote the growth of new blood vessels. However, it has also been shown to be neuroprotective against motor neuron degeneration in the context of amyotrophic lateral sclerosis (ALS) [336]. To date, the potential protective role of VEGF in HD has not yet been fully elucidated, and its use has been limited. Indeed, Ellison et al. (2013) [337] examined the effect of VGEF-165 both in vitro and in vivo. Although some protective effects were identified, it appears that higher doses of this neurotrophic factor are associated with neurotoxicity and earlier mortality. Given these concerns and the current lack of additional evidence from pre-clinical studies, it is unlikely that VEGF alone will be used as a treatment option for HD. Nevertheless, therapeutic strategies aimed at increasing trophic support in the HD brain remain an attractive avenue to treat, at least in part, this neurodegenerative disorder.

### 4.2. Autophagy Regulators

Given that autophagy dysregulation is also thought to play a role in HD neurodegeneration (see Section 1.1.4), various studies have also tested the putative beneficial effects of several autophagy regulators. Some of these studies have overexpressed modulators of the autophagy system in transgenic HD mouse models, while others have used pharmacological approaches, as outlined in the following paragraphs.

#### 4.2.1. Direct Up-Regulation of Autophagy Modulators

Ubiquilins are ubiquitin-binding proteins known to be involved in protein degradation through the ubiquitin–proteasome system (UPS). In turn, the UPS and autophagy are part of an intimately interconnected system of intracellular quality control [338,339,340]. Indeed, ubiquilins have also been shown to regulate autophagic flux by modulating mTOR signaling and lysosomal acidification [341]. Interestingly, overexpression of ubiquilin-1 in transgenic R6/2 mice resulted in delayed aggregation of mHTT in the hippocampus and increased the mean survival of these HD transgenic mice by 20%. However, ubiquilin-1 overexpression did not result in motor improvements [342].

Calpains are calcium-activated cysteine proteases, and their inhibition has been shown to promote autophagy [343]. In agreement, Menzies et al. (2015) [344] demonstrated that overexpression of the endogenous calpain inhibitor calpastatin (CAST) in N171-82Q transgenic HD mice resulted in increased autophagosome levels as well as in a significant improvement in motor function, as seen by a delay in the onset of tremors and by improved performance in the rotarod and open field tests [344].

The transcription factor EB (TFEB) regulates lysosome biogenesis and autophagy and is involved in the pathogenesis of several neurodegenerative diseases, including HD [345]. Recently, Vodicka et al. (2015) introduced a cDNA encoding TFEB with an HA tag (TFEB-HA) under the control of the neuron-specific synapsin 1 promoter into the striatum of 3-month-old zQ175 knock-in HD mice using adeno-associated virus AAV2/9. TFEB-HA overexpression resulted in increased levels of proteins involved in autophagosome/lysosome activity and reduced levels of mHTT, suggesting that TFEB expression stimulated autophagy and lysosome activity and consequently reduced mHTT in the striatum of zQ175 knock-in HD mice [346].

#### 4.2.2. Pharmacologic Modulation of Autophagy

Various compounds with the ability to modulate autophagy have now been tested in both in vitro and in vivo studies. In an initial study, N171-82Q mice treated with the rapamycin analog CCI-779 (which has more favorable pharmaceutical properties and only mild side effects) performed significantly better on the rotarod test, grip strength test, and wire maneuver test and showed a reduction in tremors [92].

Trehalose is a non-reducing disaccharide with proven neuroprotective properties related to induction of autophagy and clearance of abnormal protein aggregates [347]. Treatment of R6/2 transgenic HD mice with trehalose starting at 21 days was shown to decrease the number of mHTT aggregates, improve motor function (as assessed by the rotarod and the footprint tests), and extend the lifespan [348]. Given its lack of toxicity and high solubility, trehalose remains an interesting candidate for future clinical trials.

Rilmenidine, a well-tolerated, safe, centrally acting antihypertensive drug, has also been shown to induce autophagy in cell cultures via a pathway that is independent of mTOR. Accordingly, treatment of N171-82Q HD transgenic mice with rilmenidine reduced levels of mHTT fragments and improved grip strength, tremors, and wire maneuver performance [349]. Given these promising results, the tolerability of rilmenidine has recently been tested in 18 patients with mild to moderate HD. Although this small study demonstrated that rilmenidine is a safe and tolerable compound, no conclusive data regarding its effect on autophagy and HTT clearance were obtained [350].

Berberine, a small organic model used in Chinese traditional medicine, is also thought to induce autophagy and, consequently, reduce mHTT aggregation in vitro in cultured HEK293 cells. Moreover, N171-82Q mice treated with berberine showed significant improvements in rotarod performance and grip strength as well as increased survival [351].

AMD3100 is a G protein-coupled receptor antagonist that regulates autophagy by Zinc Finger and BTB Domain Containing 16 (ZBTB16)-mediated ubiquitination and proteasomal degradation of Beclin 1-Associated Autophagy-Related Key Regulator 14 (ATG14L). AMD3100 treatment has been shown to increase the lifespan, improve rotarod performance, and decrease hind paw clasping in N171-82Q transgenic HD mice [352].

As mentioned above, the UPS is intimately related to autophagy to ensure intracellular/protein quality control [338,339,340]. Given this, Jeon et al. (2016) injected lentivirus-expressing proteasome activator PA28γ into the striatum of YAC128 HD mice at 14–18 months of age and assessed the potential therapeutic effects of this approach 12 weeks after lentiviral administration. YAC128 mice treated with lenti-PA28γ virus showed increased peptidyl-glutamyl preferring hydrolytic (PGPH) proteasome activity, enhanced mRNA levels of BDNF, and decreased levels of ubiquitin-positive inclusions in the striatum. In addition, at the behavioral level, lenti-PA28γ virus-treated YAC128 mice showed improved motor performance, as assessed with the rotarod test [353].

Overall, it is now well accepted that dysregulation of autophagy-endolysosomal pathways is critical for the development and progression of HD neuropathology [354]. Given this, it is reasonable to speculate that restoration of autophagy pathways and the consequent promotion of selective clearance of mHTT might be effective therapeutic approaches for HD and similar neurodegenerative disorders associated with abnormal protein accumulation and aggregation.

### 4.3. Epigenetic Modulators

#### 4.3.1. Sirtuins

Sirtuins are a family of proteins that use nicotinamide adenine dinucleotide (NAD)+ as a co-factor and are therefore influenced by the ratio of NAD+/NADH [355,356]. Sirtuins are thought to play a role in autophagy by preventing acetylation of proteins (via deacetylation) [357] and exert neuroprotective functions, with SIRT1 modulating neurotrophic factors such as BDNF and interfering with the apoptotic process [355].

Some pre-clinical studies have indicated that sirtuin overexpression might be beneficial in HD models. For example, Jeong et al. (2012) [358] crossed R6/2 mice with brain-specific SIRT1 knock-out mice (BSKO) to produce a BSKO-R6/2 mouse model. BSKO-R6/2 mice were reported to have significantly smaller striatal volumes when compared to WT controls, and BSKO and R6/2 mice. Interestingly, BSKO-R6/2 animals also showed an increased number of HTT aggregates and a more severe progression of motor deficits when compared with their R6/2 counterparts. On the other hand, Sirt1-KI–R62 mice (generated by crossing R6/2 mice with transgenic mice overexpressing SIRT1 (Sirt1-KI)) displayed a significant increase in survival and a reduction in striatal atrophy when compared to R6/2 mice. Interestingly, these neuroprotective properties were primarily noted in male mice, an effect that might be related to differences in the expression levels of SIRT1 between males and females.

The neuroprotective effects of SIRT1 overexpression have also been corroborated by other studies. Indeed, crossing either N171-82Q or BACHD transgenic mice with SIRT1 overexpression transgenic mice resulted in a significant delay in the onset of motor deficits. Furthermore, in the N171-82Q model, SIRT1 overexpression was accompanied by a significant reduction in brain atrophy [359]. Moreover, treatment with the sirtuin-activating compound (STAC) SRT2104 was able to improve motor deficits in N171-82Q mice at 12, 18, and 24 weeks of age and to preserve neocortex integrity, as assessed by magnetic resonance imaging (MRI) at 22 weeks of age [360]. More recently, Lee et al. (2018) [361] demonstrated that R6/2 mice treated with β-lapachone (a natural substance with anti-inflammatory properties thought to increase SIRT1 levels) showed an improvement in rotarod performance and a decrease in paw clasping behavior [361]. Remarkably, resveratrol (a SIRT1 activator), completely restored mitochondrial membrane potential and respiration and increased PGC-1α expression and TFAM (Transcription Factor A, Mitochondrial) levels in lymphoblasts from HD patients. YAC128 mice treated for 28 consecutive days with resveratrol showed significant improvements in motor coordination and learning as well as enhanced expression of electron transport chain genes encoded by mitochondria [362].

Nevertheless, some studies have also found that sirtuin inhibition might have neuroprotective effects. Smith et al. (2014) [363] showed that treatment of R6/2 mice with the SIRT1 inhibitor selisistat significantly improved locomotor activity (although no effects on grip strength and rotarod performance were noted), reduced brain atrophy, and increased the survival of these HD transgenic mice. In agreement, treatment of R6/1 mice with nicotinamide (NAM), a known inhibitor of SIRT1, improved motor performance (i.e., as assessed with the rotarod, balance beam, and open field tests) noted at 14 weeks of age. Interestingly, NAM treatment resulted in increased BDNF levels, which might have mediated, at least in part, the beneficial effects of SIRT1 inhibition [364]. In agreement, NAM was also shown to increase levels of nicotinamide adenine dinucleotide (NAD+) and improve mitochondrial function in cellular models of HD [362].

However, to date, conflicting results regarding sirtuin activity levels in the HD brain have been reported. Indeed, Tulino et al. (2016) [365] showed decreased SIRT1 activity in two HD genetic mouse models (R6/2 and HdhQ150) [365], while a post-mortem study reported that SIRT1 mRNA levels were selectively increased in the brain of HD patients, particularly the striatum and cerebral cortex as well as in areas related to metabolic activity [366].

Although SIRT1 is the most studied sirtuin by far, the role of SIRT2 in neuroprotection has also been evaluated. For example, SIRT2 inhibition was shown to decrease mutant huntingtin toxicity by reducing HTT aggregation in a striatal neuronal model of HD [367]. In a different study using genetic HD mouse models, R6/2 and 140CAG knock-in mice treated with AK-7, a selective SIRT2 inhibitor, resulted in improved motor performance (as assessed with the rotarod test), as well as a reduction in striatal volume atrophy and the number of mHTT aggregates [368].

SIRT3, a major regulator of mitochondrial acetylome, has also been demonstrated to play a role in HD. Recently, Naia and colleagues (2021) demonstrated a consistent increased in SIRT3 levels in different models of HD and the human HD brain. Furthermore, SIRT3 overexpression enhanced mitochondrial function and antioxidant defenses, balanced the mitochondrial fission–fusion rate, and improved the anterograde transport of mitochondria along neurites, thus promoting cell survival [369].

Overall, these studies demonstrate the potential role of sirtuins in modulating neurodegenerative processes in the HD brain. Although it is still unclear whether it will be more beneficial to promote their overexpression or inhibit their function, both approaches might have their merits depending on which sirtuin-mediated pathways are affected and, potentially, the stage in the progression of the disease during which the approach is used. Conversely, since sirtuins are thought to be involved in a vast array of processes, altering their expression and/or modulating their activity levels will need to be carefully monitored and regulated accordingly.

#### 4.3.2. Histone Deacetylase (HDAC) Inhibitors and Lysine Deacetylase (KDAC) Inhibitors

HD is also associated with dysregulated histone function and a consequent disturbance in gene transcription and protein synthesis [370]. Histone acetylation is controlled by histone acetyltransferases/lysine acetyltransferases (HATs/KATs) and histone deacetylases/lysine deacetylases (HDACs/KDACs). HAT/KATs effectively “turn on” gene transcription by acetylating lysine residues located in the N-terminal of histones, whereas HDAC/KDACs “turn off” gene transcription by deacetylating these lysine residues [371]. Similar to what has been observed in other neurodegenerative diseases, the balance between histone acetylation and deacetylation is altered in HD [372,373]. Indeed, Yeh et al. (2013) [374] demonstrated that HDAC5 is increased in the caudate cells and Purkinje cells in post-mortem brains from HD patients [370]. In agreement, a few studies have evaluated the potential protective effect of HDACs in genetic mouse models of HD.

In an initial study, suberoylanilide hydroxamic acid (SAHA, a potent HDAC inhibitor) was shown to cross the blood–brain barrier and increase histone acetylation in R6/2 brains [374]. SAHA treatment (starting at postnatal week 5) significantly improved rotarod performance and reduced striatal neuronal loss, although such effects were not pronounced. However, higher SAHA doses were shown to be toxic [374], reflecting a narrow therapeutic window and indicating that HDAC inhibitors should be used cautiously.

Sodium butyrate (a modulator of class I and IIa HDACs) has also been tested in R6/2 mice [375]. Sodium butyrate extended the survival of R6/2 mice in a dose-dependent manner, improved rotarod performance, and delayed the decrease in body weight in this HD transgenic mouse model. At the neuropathological level, this HDAC inhibitor reduced brain weight loss and striatal neuronal atrophy, although no effect was observed on the number of striatal ubiquitinated NIIs [375]. Furthermore, treatment with sodium butyrate increased the acetylation of histones H3 and H4 and of the transcription factor specific protein-1 (Sp1) in R6/2 brains. The consequent improvement in transcriptional regulation resulted in increased levels of proteins involved in transcription and metabolism, which might underlie, at least in part, the protective effects of this compound [375]. Of note, sodium butyrate and the pan-lysine HDACI trichostatin A (TSA) were also shown to ameliorate mitochondrial function in immortalized striatal (STHdh) cells and striatal neurons from YAC128 mice [376]. More recently, sodium butyrate was shown to promote the activity of pyruvate dehydrogenase, thus helping to counteract HD-related mitochondrial metabolism deficiency and improve motor function in YAC128 mice. Such outcome suggests that modulation of mitochondrial function through DNA expression modifications can be a promising strategy for the treatment of HD [377].

Thomas et al. (2008) [378] also showed significant amelioration of hind limb clasping, rotarod performance, and general locomotor ability in R6/2 mice treated with the selective HDAC inhibitor 4b (HDACi 4b). Moreover, HDACi 4b-treated R6/2 mice also showed a reduction in striatal atrophy and ventricular enlargement as compared to their vehicle-treated HD counterparts [378]. Similar results were also observed in N171-82Q HD transgenic mice treated with HDACi 4b. Indeed, HDACi 4b treatment resulted in significant locomotor and cognitive improvements (as assessed with the rotarod/open field tests and the T-maze test, respectively) [379]. Furthermore, N171-82Q HD transgenic mice treated with the specific HDAC3 inhibitor RGFP966 also showed significant locomotor improvements on the rotarod and in the open field test as well as reduced neuronal atrophy. However, RGFP966 treatment did not affect the number of mHTT aggregates [380].

More recently, Chopra and colleagues (2016) [381] showed that treatment with LBH589 (panobinostat, a non-selective HDAC inhibitor able to cross the blood–brain barrier) significantly reduced striatal neuronal atrophy in female R6/2 mice and CAG140 KI mice. Regarding locomotor activity, panobinostat only improved motor performance (as assessed with the accelerating rotarod test) in CAG140 KI mice but not in R6/2 mice [381].

Despite the beneficial effects reported following HDAC inhibition and summarized above, not all HDACs have been shown to result in an amelioration of HD-related phenotypes. For example, Bobrowska et al. (2011) [382] showed that HDAC6 inhibition did not significantly impact the phenotype of R6/2 mice. Furthermore, issues with bioavailability with some of the currently available HDACs and their potential neurotoxic effects complicate their use in the clinic [371]. Given this, further research aimed at improving bioavailability and optimizing ideal doses and therapeutic windows is therefore warranted before these can be considered valid approaches for the treatment of human HD patients.

### 4.4. Nanotechnology and Nanoparticles

An important obstacle that limits the use of many potential therapeutic approaches for the treatment of HD and other neurodegenerative disorders is the limited ability of many compounds to cross the blood–brain barrier. Furthermore, considerable inflammation and damage to the brain (and the blood–brain barrier itself) resulting from the actual neurodegenerative processes can significantly impact the efficacy of delivery methods. The use of nanotechnology to safely deliver neuroprotective agents to affected regions of the brain has emerged as a potential strategy to overcome these issues [383,384,385,386,387,388].

Along these lines, recent studies by Ramachandran and Thangarajan (2016, 2018) [389,390] demonstrated that solid lipid nanoparticles loaded with thymoquinone (an antioxidant and anti-inflammatory compound) were superior when compared with administration of a thymoquinone suspension in improving the neuropathological features (i.e., oxidative stress and excitotoxic markers) and behavioral phenotypes (muscle strength, rigidity, movement, and memory performances) of 3-NP-lesioned rats. Overall, these studies showed that with nanoformulation, lower doses of thymoquinone are required to achieve therapeutic effects [389,390]. Similarly, Bhatt and colleagues (2015) [391] used solid lipid nanoparticles to deliver rosmarinic acid (a caffeic acid derivative with antioxidant and anti-inflammatory properties) into the brain of 3-NP-lesioned rats. Rosmarinic acid-loaded nanoparticles significantly improved behavioral abnormalities and reduced oxidative stress in 3NP-treated rats, particularly when delivered intranasally (as compared with intravenous delivery) [391].

Although the use of nanotechnology and nanoparticles has just recently started to be explored in the context of HD, it offers an exciting and promising avenue to not only deliver safe and selective antioxidant and anti-inflammatory agents to specific regions of the brain but to also act to facilitate genomic (i.e., personalized) medicine [392]. Nevertheless, this exciting technology still requires further refinement and optimization before being considered a practical option for delivering drugs and other biomolecules in the clinical setting. In particular, patterns of the brain distribution of the nanoparticles and their cargos, the metabolism of different nanocarriers, and the practical implications of administering these compounds will need to be fully understood and considered [392]. Future studies are thus warranted to further elucidate the mechanisms and utility of this technology in the context of neurodegenerative diseases, in general, and HD, in particular.

### 4.5. Stem Cell Treatment

Stem cell research has gained increased attention over recent decades [393], and stem cell therapies have been considered for HD (see reviews, [161,394]), under the assumption that new neurons can replace degenerating cells in the affected brain regions and, consequently, ameliorate the disease profile [395]. Numerous in vitro studies have been conducted to characterize several types of pluripotent stem cells, including embryonic stem cells, mesenchymal cells, and neural stem cells, and some have shown great potential as putative therapeutic options for neurodegenerative conditions [396,397,398]. Although a comprehensive review of all studies that have tested stem cell therapies in HD models is outside of the scope of the present review, the following paragraphs will provide a brief overview of studies that have been conducted in genetic HD mouse models. A recent and more comprehensive review on this topic can be found elsewhere [399].

In an initial study, Dunnett and colleagues (1998) [400] evaluated the survival and differentiation of striatal grafts and their impact in R6/2 mice. Striatal tissue was harvested from wild-type mouse embryos at embryonic day 14 and bilaterally injected into the striatum of 10-week-old R6/2 mice. Although the grafts survived and appeared to have successfully integrated within the host brain, the beneficial effects on locomotion (as assessed with the open field test) were minimal [400]. This lack of effect might be due, at least in part, to the fact that transplantation was performed during an advanced symptomatic stage, and therefore additional studies aimed at determining the efficacy of striatal embryonic stem cell grafting during the early stages of the disease are warranted. In a subsequent study, van Dellen and colleagues (2001) transplanted wild-type embryonic cortical tissue (at embryonic day 16) into the anterior cingulate cortex of neonatal R6/1 mice [401]. Although no improvements were noticed in the horizontal rod test, a delay in the onset of paw clasping was observed, suggesting that this cortical region plays a role in developing some of the motor deficits seen in R6/1 HD transgenic mice [401].

C17.2 neural stem cells have the potential to adopt a neuronal phenotype and have been previously shown to be effective in models of neurodegenerative disorders [402,403]. Given this, Yang and colleagues (2008) [404] also assessed the potential beneficial effects of transplanting C17.2 neural stem cells in R6/2 mice. Mice received a diet enriched with trehalose (thought to decrease aggregate formation; see Section 4.2) starting at 3 weeks of age, and at 8 weeks, C17.2 stem cells were transplanted into the lateral ventricles. Trehalose treatment combined with C17.2 stem cell grafting was shown to significantly reduce ubiquitin-positive aggregation in the striatum and lateral ventricle enlargement, delay the onset of clasping, improve motor function (as assessed by the footprint test), and extend the R6/2 lifespan by up to 26.3% [404]. Together, these findings provide support to further explore the potential of combined pharmacological and stem cell therapies for the treatment of HD.

The use of mesenchymal stem cells (MSCs) derived from the umbilical cord offers an attractive alternative for transplantation as the umbilical cord is a non-controversial and inexhaustible source of stem cells that can be harvested at a low cost. With this in mind, Fink et al. (2013) [405] isolated MSCs from the umbilical cord of 15-day gestation mouse pups and transplanted them into 5-week-old R6/2 mice at either a low passage or high passage. No effects on motor deficits were observed, although a transient improvement in a spatial memory task was seen. However, grafting of umbilical cord-derived MSCs appeared to alleviate some of the neuropathological features seen in R6/2 mice, with high-passage MSCs providing more benefit than low-passage MSCs [405]. Striatal grafting of bone marrow-derived MSCs has also been tested in R6/2 mice, and similar results have been reported [401]. Indeed, while R6/2 mice transplanted with low-passage MSCs did not show significant locomotor improvements, transplantation of high-passage MSCs resulted in improved locomotion (as assessed with the rotarod test) and delayed limb clasping behavior (until 10 weeks of age). Nevertheless, both low- and high-passage MSC grafting has a moderate effect on the Morris water maze test. Despite these promising results, the observed behavioral improvements were not sustainable over time and as the disease progressed [406], which might be due, at least in part, to the fast disease progression seen in R6/2 mice.

MSCs derived from the human amniotic membrane are thought to have immunomodulatory properties and a positive impact on inflammatory processes. Given this, their applicability for the treatment of HD has recently been tested in the R6/2 transgenic mouse model [407]. R6/2 mice treated with MSCs derived from the human amniotic membrane showed improved motor performance (assessed with the rotarod and the open field tests and by a reduced clasping response). At the neuropathological level, MSC treatment resulted in reduced striatal atrophy. Overall, these results indicate that stem cell therapies aimed at modulating the inflammatory response might be quite beneficial in alleviating some of the features of HD [407]. The use of human embryonic stem cell-derived neural stem cells also constitutes a potential option for transplantation studies. Reidling et al. (2018) [408] transplanted good manufacturing practice (GMP)-grade human embryonic stem cell-derived neural stem cells into the striatum of both R6/2 and Q140 HD knock-in mice. Transplants were shown to integrate into the existing neuronal circuitry, rescue synaptic alterations, and improve motor deficits (as assessed with the pole test, rotarod task, and grip strength) in R6/2 mice and to ameliorate late-stage cognitive impairment in Q140 knock-in mice. Furthermore, human embryonic stem cell-derived neural stem cells also reduced mHTT accumulation and promoted BDNF expression in both HD models [408].

A few studies have also evaluated the effects of stem cell therapies in transgenic full-length mouse models of HD. Dey et al. (2010) [409] studied the effects of bone marrow mesenchymal cell transplantation in YAC128 HD mice. Bone marrow mesenchymal cells were harvested from the femur of mice genetically engineered to overexpress either BDNF or nerve growth factor (NGF) and were implanted into the striatum of 4-month-old YAC128 mice. YAC128 mice transplanted with either BDNF- or NGF-overexpressing striatal grafts displayed significant motor improvements (as assessed with the rotarod test), with BDNF-overexpressing mice showing a more sustained response when compared to their NGF-overexpressing counterparts. Furthermore, BDNF-overexpressing grafts were shown to decrease neuronal loss, further highlighting the therapeutic effect of inducing BDNF-mediated trophic support in the HD brain (see Section 4.1.1). In a more recent study, Al-Gharaibeh et al. (2017) [410] examined the potential effects of intrastriatal transplantation of induced pluripotent stem cell-derived neural stem cells (iPS-NSCs) in YAC128 mice. Mouse adenovirus-generated iPSCs were differentiated into neural stem cells in vitro and bilaterally transplanted into the striatum of 10-month-old YAC128 mice. iPS-NSC transplantation resulted in improvement in motor deficits (as assessed with the rotarod test). At the neuropathological level, iPS-NSC transplantation reduced striatal neuronal loss and increased striatal levels of both BDNF and TrkB [410].

Overall, stem cell-based therapies remain a promising avenue for the treatment of neurodegenerative diseases such as HD. However, before this could become a viable option for HD-afflicted individuals, it is imperative to identify the sources of stem cells that yield the best possible outcomes and optimize the various available protocols. As such, additional pre-clinical studies using rodent and possibly non-human primate models of HD are warranted to further test these approaches.

### 4.6. Genetic Manipulations

The fact that HD is a genetic disorder makes it an attractive candidate for gene therapy. Specifically, the selective suppression of mHTT expression is expected to delay the onset and mitigate the severity of the disease, by preventing the toxic gain of function of the mutated protein without inducing the loss of function of its normal counterpart [30]. Given this, the efficacy of small interfering RNAs (siRNAs) directed against the HD gene in repressing the expression of the mutant protein and alleviating the disease phenotype has been tested in R6/2 mice [411]. Intracerebroventricular administration of siRNAs at postnatal day 2 significantly extended the lifespan of R6/2 mice by more than 2 weeks and improved their motor deficits (as assessed by rotarod and open field tests as well as paw clasping behavior). At the neuropathological level, siRNAs reduced striatal atrophy (as evidenced by a decrease in lateral ventricle enlargement) and the number of striatal NIIs near the injection site [411]. In agreement with these initial findings, a subsequent study also demonstrated that a single striatal administration of an siRNA targeting mutant huntingtin could silence the mutant gene and consequently reduce neuropathological signs and behavioral abnormalities observed in a rapid-onset viral transgenic HD mouse model [412]. Subsequent studies using fibroblasts from human HD patients have provided promising results, suggesting that allele-specific siRNA treatment may be clinically feasible and result in the treatment of a large number of HD-affected individuals [413,414].

siRNA technology has also been used to perform large-scale drug screens. Indeed, Jimenez-Sanchez and colleagues (2015) performed a “druggable genome” siRNA screen using human cell cultures, followed by validation in Drosophila and zebrafish HD models. Through this screening, glutaminyl cyclase (QPCT) was identified as a potential therapeutic target for HD, as its inhibition resulted in decreased abnormal protein aggregation [415].

Of note, many other in vitro studies have been conducted using different types of RNA interference (RNAi)/microRNA with encouraging results, particularly when it comes to reducing mutant huntingtin aggregation (see, for example, [416,417]). In addition, some studies have indicated that these in vitro effects can be replicated in HD mouse models. For example, N171-82Q HD mice treated with non-specific RNAi showed improved motor performance on the rotarod and a 75% decrease in the amount of huntingtin expressed. Though not significant, N171-82Q HD mice treated with non-allele-specific RNAi had increased survival, with >75% of animals living until 20 weeks of age (i.e., time of sacrifice) [418]. More recently, injection of an adeno-associated viral vector expressing a microRNA targeting human HTT (AAV5-miHTT) into zQ175 knock-in HD mice resulted in reduced HTT aggregation in the striatum and cortex. Furthermore, this genetic manipulation also significantly improved the rotarod performance and survival (by 4 weeks) of R6/2 mice [237]. Of note, the beneficial effects of RNAi-based therapies can also be translated to larger HD animal models, including minipigs and rhesus macaques [236,419].

A different gene therapy approach was tested by Huang and collaborators (2007) [420], who generated a high-capacity adenoviral (HC-Ad) vector expressing a short hairpin RNA (shRNA) targeted to exon 1 of the HD gene. Five-week-old R6/2 mice received striatal injections of this shRNA-containing viral vector, and a significant reduction in the number of NIIs was noted [420]. However, this was a pilot study as no data on the effects of this type of gene therapy on survival, behavioral deficits, and other neuropathological features were reported. Although shRNAs are generally considered toxic due to the saturation of the miRINA biogenesis pathway [421,422], when compared with siRNAs, shRNAs may be less toxic due to the fewer “off-target” effects and the longer-lasting approach. However, there are still technical difficulties when it comes to controlling the expression of shRNA [423].

More recently, Keeler et al. (2016) evaluated the effects of an adeno-associated virus 9 (AAV9)-green fluorescent protein (GFP)-miRHtt vector on HTT mRNA levels in striatal neurons of Q140/Q140 knock-in mice. Mice received bilateral striatal injections of AAV9-GFP-miRHtt or AAV9-GFP at 6 or 12 weeks, and striata were evaluated at 6 months of age for levels of Htt mRNA. Intrastriatal infusion of AAV9-GFP-miRHtt significantly reduced striatal HTT mRNA expression by approximately 50%, through a partial reduction in the number of copies of mutant HTT mRNAs per cell [424]. In a subsequent study, Pfister and colleagues (2017) compared the effects of a mir-155-based artificial miRNA under the control of either the chicken β-actin promoter or the human U6 promoter in YAC128 mice. This artificial miRNA reduced the levels of human HTT mRNA by approximately 50%. However, the U6, but not the CβA, promoter resulted in artificial supraphysiologic levels of miRNA. Of note, 6 months post-injection, YAC128 mice treated with scAAV9-CβA-mir-155-HTT were indistinguishable from controls, whereas those that received scAAV9-U6-mir-155-HTT showed behavioral abnormalities and striatal damage. This study indicates that the CβA promoter can provide an effective and safe dose of a human huntingtin miRNA [425].

Another genetic therapeutic approach is the use of the clustered regulatory interspaced short palindromic repeats (CRISPR)/CRISPR associated protein 9 (Cas9) system as a gene editing tool [160,426]. In the case of HD, preliminary studies using patient-derived HD fibroblasts demonstrated a complete inactivation of mHTT [427,428]. In the Q140 knock-in HD mouse model, CRISPR/Cas9 reduced mHTT and attenuated both behavioral and neuropathological features, although no effect on survival was noted [429]. Of note, a recent modification of the CRISPR/Cas9 tool aimed at improving safety and reducing the potential for off-target effects has been used to edit the HD gene. Using this approach, protein aggregation and neuronal dysfunction were reduced in HD transgenic mice [430]. However, as with other therapies, the development of CRISPR Cas9-based therapies to treat HD will require further methodological optimization and thorough elucidation of off-target effects [431,432,433].

Despite these promising results, most of the genetic approaches for the treatment of HD are still in their infancy, and potential barriers to their use (e.g., delivery mode, accessibility, and tolerability, among others) still require optimization before these can be used in large clinical trials. Nevertheless, these are, by far, the treatment modalities with the greatest potential to modify disease outcomes and potentially cure this devastating genetic neurodegenerative disorder [160].

## 5. Conclusions

Since the discovery of the mutation responsible for HD [11], much has been learned regarding the mechanisms that underlie the specific neurodegeneration characteristic of this disease [30]. In addition, the availability of HD transgenic mouse models has facilitated and accelerated the pre-clinical evaluation of putative therapeutic agents [130,131,434]. However, while most of these therapies have generally been successful in pre-clinical studies, the benefits observed in clinical trials involving HD patients have been somewhat limited. These discrepancies likely arise from several factors including failure to conduct pre-clinical studies using concentrations, time windows, and models relevant to the clinical setting. In addition, it is also important to note that, given the multi-factorial nature of HD [30], a combination of drugs targeting different intracellular, epigenetic, and genetic factors is likely to result in better outcomes for the mitigation of HD symptoms. Surprisingly, however, there are only a limited number of active clinical trials currently testing the potential beneficial effects of combining two or more treatment strategies.

While the number of undergoing clinical trials demonstrates the efforts of a growing scientific and medical community in the search for a disease-modifying treatment or cure for this devastating neurodegenerative disorder, a few recommendations are warranted. To produce comparable results across different sites and studies, future clinical trials should incorporate methods to decrease placebo and bias effects, employ standardized and validated rating scales, and conduct rigorous statistical analyses [435]. Moreover, variables such as the number of CAG repeats can greatly influence the results of clinical trials [436] and therefore should also be considered. Finally, the development of more refined tests that are sensitive to the longitudinal cognitive changes in HD and selecting appropriate cognitive outcomes [437] will further aid in the design of clinical trials aimed at ameliorating the cognitive decline associated with this disorder. Overall, these considerations may benefit the design of more robust pre-clinical and clinical studies.

Finally, the design of new clinical trials that test the tolerability and efficacy of strategies with the potential to modify the course of the disease (such as some of the epigenetic, genetic, and stem cell-based strategies described in Section 4) is warranted and highly anticipated. The promising results that have been obtained with these approaches in the pre-clinical setting make them excellent candidates for future clinical trials. With further refinement of these strategies, a treatment for this devastating disorder will be, finally, within our reach.

## Figures and Tables

**Table 1:** Clinical trials for Huntington’s disease (HD) currently active and/or recruiting participants registered at Clinicaltrials.gov (accessed on 2 July 2021).

Intervention (Mechanism)	CT Identifier	Clinical Trial	Stage	Phase	Allocation	Masking	Population	Period
**Dopaminergic Modulation**								
Drug: Tetrabenazine	NCT02509793	A Pilot Study Assessing Impulsivity in Patients with Huntington’s Disease on Xenazine (Tetrabenazine)	Recruiting	Phase IV	Single Group Assignment	Open Label	20	August 2018–July 2023
Drug: Deutetrabenazine	NCT04301726	Efficacy of Deutetrabenazine to Control Symptoms of Dysphagia Associated with HD	Not yet recruiting	PhaseI	Randomized	Triple	48	September 2020–December 2022
	NCT04713982	Impact of Deutetrabenazine on Functional Speech and Gait Dynamics in Huntington Disease	Recruiting	Phase II/III	N/A	Open Label	30	July 2021–February 2024
Drug: Valbenazine	NCT04102579	Efficacy, Safety, and Tolerability of Valbenazine for the Treatment of Chorea Associated with Huntington Disease (KINECT-HD)	Recruiting	Phase III	Randomized	Quadruple blind	120	November 2019–September 2021
Drug: Risperidone	NCT04201834	Study to assess the safety and benefit of risperidone for the treatment of chorea in Huntington’s disease	Recruiting	Phase II	N/A	Open Label	12	August 2020–August 2022
**Glutamatergic Modulation **								
Drug: Dextromethorphan/ quinidine	NCT03854019	Evaluating the Efficacy of Dextromethorphan/Quinidine in Treating Irritability in Huntington’s Disease	Recruiting	Phase III	Randomized	Quadruple blind	22	April 2019–December 2021
**Synaptic Modulation**								
Drug: Neflamapimod	NCT03980938	Within Subject Crossover Study of Cognitive Effects of Neflamapimod in Early-Stage Huntington Disease	Recruiting	PhaseII	Randomized	Quadruple blind	16	July 2019–July 2020
**BDNF Levels Modulation **								
Drug: Pridopidine	NCT04556656	Pridopidine’s Outcome on Function in Huntington Disease, PROOF- HD	Recruiting	Phase III	Randomized	Quadruple blind	480	October 2020–April 2023
**Mitochondrial Function and Biogenesis**								
Drug: Fenofibrate	NCT03515213	Safety and Efficacy of Fenofibrate as a Treatment for Huntington’s Disease	Active, not recruiting	Phase II	Randomized	Triple blind	20	April 2017–August 2021
Drug: Triheptanoin oil	NCT02453061	A Comparative Phase 2 Study Assessing the Efficacy of Triheptanoin, an Anaplerotic Therapy in Huntington’s Disease	Active, not recruiting	Phase II	Randomized	Quadruple blind	100	June 2015–December 2020
Drug: Metformin	NCT04826692	Study to Assess the Effect of Metformin, an Activator of AMPK, on Cognitive Measures of Progression in Huntington’s Disease Patients	Not yet recruiting	Phase III	Randomized	Double	60	September 2021–August 2024
**Aggregate Inhibition**								
Drug: Nilotinib	NCT03764215	Nilotinib in Huntington’s Disease	Recruiting	Phase I	Sequential Assignment	Open Label	10	November 2018–November 2020
**Stem Cell Therapies**								
Biological: Cellavita	NCT02728115	Safety Evaluation of Cellavita HD Administered Intravenously in Participants with Huntington’s Disease	Active, not recruiting	Phase I	Non-Randomized	Open Label	6	October 2017–December 2023
	NCT03252535	Dose-response Evaluation of the Cellavita HD Product in Patients with Huntington’s Disease	Active, not recruiting	Phase II	Randomized	Triple blind	35	January 2018–April 2022
	NCT04219241	Clinical Extension Study for Assessing the Safety and Efficacy of the Intravenous Administration of Cellavita-HD in Huntington’s Disease Patients.	Active, not recruiting	Phase II/III	N/A	Open Label	35	January 2020–April 2022
**Genetic Manipulations **								
Drug: RO7234292 (RG 6042, IONIS-HTTRx) intrathecal injection	NCT03842969	An Open-Label Extension Study to Evaluate Long-Term Safety and Tolerability of RO7234292 (RG6042) in Huntington’s Disease Patients Who Participated in Prior Roche and Genetech Sponsored Studies	Recruiting	Phase III	Randomized	Open Label	950	April 2019–June 2024
	NCT04000594	A Study to Investigate the Pharmacokinetics and Pharmacodynamics of RO7234292 (RG6042) in CSF and Plasma, and Safety and Tolerability Following Intrathecal Administration in Patients with Huntington’s Disease	Recruiting	Phase I	Non-Randomized	Open Label	20	September 2019–December 2021
Genetic: intra-striatal rAAV5-miHTT	NCT04120493	Safety and Proof-of-Concept (POC) Study With AMT-130 in Adults with Early Manifest Huntington Disease	Recruiting	Phase I/II	Randomized	Triple blind	26	September 2019–May 2026
Genetic: Intraparenchymal rAAV1-(mi)RNA HTT	NCT04885114	Safety and Tolerability Study With VY-HTT01, in Adults with Early Manifesting Huntington’s Disease	Not yet recruiting	Phase I	Randomized	Open Label	22	July 2021–December 2024
**Brain Stimulation**								
Deep Brain Stimulation	NCT02535884	Deep Brain Stimulation of the Globus Pallidus (GP) in Huntington’s Disease (HD)	Recruiting	N/A	Randomized	Quadruple blind	50	July 2014–December 2022
	NCT04244513	Deep Brain Stimulation Treatment for Chorea in Huntington’s Disease	Recruiting	N/A	Randomized	Quadruple	40	February 2020–June 2022
Non-invasive Brain Stimulation	NCT04429230	Efficacy of non-invasive brain stimulation via Transcranial pulsed current stimulation (tPCS) in patients of Huntington’s disease	Not yet recruiting	N/A	Randomized	Double	15	June 2021–December 2022
**Physical Activity**								
Behavioral: Physical activity	NCT03344601	Physical Activity and Exercise Outcomes in Huntington’s Disease (PACE-HD)	Active, not recruiting	N/A	Randomized	Open Label	116	February 2018–August 2020
Behavioral: Adapted Physical Activity program	NCT04917133	Adapted Physical Activity Effect on Abilities and Quality of Life of Huntington Patients and Relatives During Rehab Stay (HUNT’ACTIV)	Not yet recruiting	N/A	Randomized	Open Label	32	June 2021–January 2023
**Dietary Supplement **								
Dietary Supplement: Melatonin	NCT04421339	Melatonin for Huntington’s Disease (HD) Gene Carriers with HD Related Sleep Disturbance—a Pilot Study	Recruiting	N/A	Randomized	Double	20	June 2020–July 2021
Drug: combined oral thiamine with biotin	NCT04478734	Trial of the Combined Use of Thiamine and Biotin in Patients with Huntington’s Disease (HUNTIAM)	Not yet recruiting	Phase II	Randomized	Open Label	24	April 2021–August 2022
**Antibody Therapy**								
Drug: ANX005	NCT04514367	An Open Label Study of ANX005 in Subjects With, or at Risk for, Manifest Huntington’s Disease	Recruiting	Phase II	N/A	Open Label	24	August 2020–June 2022
**Treatment Regimen**								
Drugs: Deutetrabenazine, Risperidone, Zoloft and Idebenone (depending on demand and symptom)	NCT04071639	Symptomatic Therapy for Patients with Huntington’s Disease	Recruiting	Phase I	Non-Randomized	Open Label	60	March 2020–December 2024

## Data Availability

No new data were created or analyzed in this study. Data sharing is not applicable to this article.
